# Trends in genetic diversity and the effect of inbreeding in American Angus cattle under genomic selection

**DOI:** 10.1186/s12711-021-00644-z

**Published:** 2021-06-16

**Authors:** Emmanuel A. Lozada-Soto, Christian Maltecca, Duc Lu, Stephen Miller, John B. Cole, Francesco Tiezzi

**Affiliations:** 1 Department of Animal Science, North Carolina State University, Raleigh, NC 27607 USA; 2 Angus Genetics Inc, St. Joseph, MO 64506 USA; 3 Animal Genomics and Improvement Laboratory, Henry A. Wallace Beltsville Agricultural Research Service, USDA, Beltsville, MD 20705 USA

## Abstract

**Background:**

While the adoption of genomic evaluations in livestock has increased genetic gain rates, its effects on genetic diversity and accumulation of inbreeding have raised concerns in cattle populations. Increased inbreeding may affect fitness and decrease the mean performance for economically important traits, such as fertility and growth in beef cattle, with the age of inbreeding having a possible effect on the magnitude of inbreeding depression. The purpose of this study was to determine changes in genetic diversity as a result of the implementation of genomic selection in Angus cattle and quantify potential inbreeding depression effects of total pedigree and genomic inbreeding, and also to investigate the impact of recent and ancient inbreeding.

**Results:**

We found that the yearly rate of inbreeding accumulation remained similar in sires and decreased significantly in dams since the implementation of genomic selection. Other measures such as effective population size and the effective number of chromosome segments show little evidence of a detrimental effect of using genomic selection strategies on the genetic diversity of beef cattle. We also quantified pedigree and genomic inbreeding depression for fertility and growth. While inbreeding did not affect fertility, an increase in pedigree or genomic inbreeding was associated with decreased birth weight, weaning weight, and post-weaning gain in both sexes. We also measured the impact of the age of inbreeding and found that recent inbreeding had a larger depressive effect on growth than ancient inbreeding.

**Conclusions:**

In this study, we sought to quantify and understand the possible consequences of genomic selection on the genetic diversity of American Angus cattle. In both sires and dams, we found that, generally, genomic selection resulted in decreased rates of pedigree and genomic inbreeding accumulation and increased or sustained effective population sizes and number of independently segregating chromosome segments. We also found significant depressive effects of inbreeding accumulation on economically important growth traits, particularly with genomic and recent inbreeding.

**Supplementary Information:**

The online version contains supplementary material available at 10.1186/s12711-021-00644-z.

## Background

It has been more than two decades since the idea of using genomic markers to increase the prediction accuracy of an animal’s genetic value was first laid out [1]. Since then, genomic selection (GS) has been incorporated into breeding programs for a wide array of livestock species and has dramatically increased the rate of genetic progress, mainly thanks to improved prediction of breeding values and shortened generation intervals [2–4]. In dairy cattle, genomic selection has increased the improvement rate for many economically important traits, particularly for lowly heritable traits associated with longevity and health [3, 4]. Differences between the dairy and beef industries, such as differences in the ease of phenotype collection on the selection candidates, the prevalence of sex-limited traits, and the use of crossbreeding, could explain the slower adoption of genomic selection strategies in beef compared to dairy cattle breeds [2]. Nonetheless, routine genomic evaluations have been implemented in the American Angus breed since 2009, and over 900,000 animals have been genotyped to date.

In the early days of GS, there was speculation about its impact on the accumulation of inbreeding and genetic diversity. Schaeffer [5] hypothesized that reducing generation intervals due to accurately predicting breeding values at birth could increase inbreeding. Similarly, the ability to evaluate selection candidates in a wide array of environments was speculated to decrease specialized populations and decrease effective population sizes (${N}_{e}$). Daetwyler [6] theorized that the ability to account for Mendelian sampling with genomic information would lead to reductions in yearly ($\Delta {\text{F}}_{\text{yearly}}$) and generational ($\Delta {\text{F}}_{\text{gen}}$) rates of inbreeding by decreasing the co-selection of sibs. In dairy cattle, the reality has been that yearly and generational rates of pedigree and genomic inbreeding and coancestry have increased in Dutch-Flemish [7], French [8], and North American [9] populations of the Holstein–Friesian dairy breed.

In addition to the loss of genetic diversity associated with high levels of inbreeding [10], the accumulation of inbreeding in the population can cause an unfavorable increase or decrease in the mean phenotypic value of individuals for a particular trait, a phenomenon called inbreeding depression [11]. Quantitative genetics theory states that the reduction in the population mean due to inbreeding is due to increased homozygosity at loci where the heterozygote differs from the average value of the homozygotes, which happens when dominance is at play [11]. Evidence amassed from experiments conducted in plant and animal populations points to increased homozygosity at loci harboring deleterious variants as a contributing factor to inbreeding depression [12]. Inbreeding depression has been documented in beef cattle for growth and reproduction traits. Carolino and Gama [13] found that an increase in direct and maternal pedigree inbreeding decreased calf birth weight and weight at 3 and 7 months of age. Direct inbreeding also reduced the weight at 12 months of age and increased the age at first calving and calving intervals. Pereira et al. [14] found similar detrimental effects of direct pedigree inbreeding on growth with decreased weaning weight and on post-weaning growth and reproduction with increased first calving interval and days open.

The availability of genomic information has allowed us to capture Mendelian sampling variation and determine the realized inbreeding load of an animal, instead of only the expected load based on pedigree relationships. In addition, it permits the analysis of animals/populations with incomplete or missing pedigree records. Genomic information can be used to estimate inbreeding depression due to marker or segment-based inbreeding. For example, Reverter et al. [15] found a consistently negative impact of marker and segment-based genomic inbreeding on yearling body weight in tropical cattle across marker panels of varying density.

Not all inbreeding is the same or is expected to have depressive effects on fitness. The ‘age’ of inbreeding is expected to moderate its effects, with older inbreeding having more time for purifying selection to act upon it and purge the population’s detrimental alleles. From this standpoint, pedigree and genomic inbreeding can be split into inbreeding age classes that permit the comparison of inbreeding depression caused by recent and old inbreeding. For example, recent pedigree and genomic inbreeding are more detrimental to milk and milk components yield, heifer and cow reproduction, and health traits than ancient inbreeding in Dutch [16] and Canadian [17] Holstein-Friesians. To the best of our knowledge, similar studies have not yet been performed in beef cattle.

The aims of our study were to (1) characterize the American Angus population in terms of pedigree and genomic inbreeding levels, (2) determine the magnitude and direction of changes to the rate of inbreeding, generation intervals, effective population size, and the effective number of chromosomal segments since the implementation of genomic evaluations, and (3) quantify the effects of recent and ancient inbreeding that affect reproductive and growth traits in Angus cattle.

## Methods

### Animals and data

All data for this study were provided by the American Angus Association (AAA). In total, 569,364 American Angus individuals registered with the AAA born between 1969 and 2019 were used. Pedigree data was obtained from Angus Genetics, Inc. (St. Joseph, MO) and contained 1,372,734 animals, including 25,692 founders. Pedigree statistics, including number of complete generations (NCG), complete generation equivalents (CGE), and pedigree completeness index (PCI), were obtained using the optiSel R package [18]. The CGE represents the sum of ${(\frac{1}{2})}^{n}$ known ancestors of each individual, where $n$ is the number of generations between an individual and its ancestor. The PCI of an individual represents the harmonic mean of the pedigree completeness of its parents, calculated according to MacCluer [19]. These pedigree statistics were used to discriminate individuals based on pedigree completeness to reduce potential bias in estimating pedigree inbreeding coefficients. Individuals with a NCG smaller than three complete generations, CGE less than ten equivalent generations, and/or a PCI that takes four generations into account lower than 0.90 were discarded from further analysis. In total, 19,532 animals that did not meet pedigree completeness requirements were removed from the dataset. The remaining animals had an average NCG of 10.74 complete generations, an average CGE of 21.21 equivalent generations, and an average PCI of 1.00.

Animals were genotyped with one of several genotyping platforms used over time at the AAA, including GGP HD, GGP HD150K, GGP LD (https://genomics.neogen.com/pdf/ag337_ggp_productportfoliobrochure.pdf), HD50K (https://www.zoetisus.com/animal-genetics/beef/hd-50k/index.aspx), i50K (https://www.zoetisus.com/animal-genetics/media/documents/i50k-00001_50k-sellsheet.pdf), and Angus GS v.1, AnGS hereafter, (https://www.neogen.com/neocenter/press-releases/angus-genetics-neogen-introduce-angus-gs-genomic-profile/). Single nucleotide polymorphisms (SNPs) that met one of the following criteria were excluded from the process: (1) SNPs on sex chromosomes; (2) a Mendelian inconsistency higher than 2%; (3) a call rate lower than 90%; and (4) a minor allele frequency lower than 0.1%. Animals with a call rate lower than 90% were removed. Taking advantage of overlapping SNPs between arrays and implementing a multi-step imputation process, all animals were imputed using the FImpute v3.0 program [20] to an ultimate SNP panel that consisted of 92,941 SNPs, referred to as C92K hereafter, and used in the analyses, hereafter.

For this analysis, further quality control procedures were performed, including removing animals with a call rate lower than 99% and SNPs with a call rate lower than 95% and/or a minor allele frequency lower than 0.1%. After quality control procedures, 549,407 individuals and 89,206 SNP remained for further analysis.

### Inbreeding measures

Several measures were used to quantify the amount and determine the age of inbreeding that has accumulated in the AAA population over time, including pedigree and genomic based inbreeding coefficients, with the latter including inbreeding based on the genomic relationship matrix (GRM), runs of homozygosity (ROH), and homozygous-by-descent (HBD) segments.

#### Pedigree-based measures

Total pedigree inbreeding (${\text{F}}_{\text{PED}}$) was estimated using the SNP1101 software [21]. In order to decompose ${\text{F}}_{\text{PED}}$ into age classes, we also calculated pedigree inbreeding using SNP1101 based on the first 3 (${\text{F}}_{\text{PED}3}$), 4 (${\text{F}}_{\text{PED}4-3}$), 5 (${\text{F}}_{\text{PED}5-4}$), 6 (${\text{F}}_{\text{PED}6-5}$), 7 (${\text{F}}_{\text{PED}7-6}$), and 8 (${\text{F}}_{\text{PED}8-7}$) ancestral generations, where the inbreeding coefficients for ${\text{F}}_{\text{PED}4-3}$, ${\text{F}}_{\text{PED}5-4}$, ${\text{F}}_{\text{PED}6-5}$, ${\text{F}}_{\text{PED}7-6}$, and ${\text{F}}_{\text{PED}8-7}$ were calculated as the difference between the inbreeding coefficients of that age class and the preceding age class (i.e., ${\text{F}}_{\text{PED}4-3}={\text{F}}_{\text{PED}4}-{\text{F}}_{\text{PED}3}$). In addition, a class based on inbreeding accumulated nine generations ago or more distantly in the past was also created (${\text{F}}_{\text{PED}9+}$).

#### Genomic-based measures

Genomic inbreeding coefficients were calculated using marker-by-marker (${\text{F}}_{\text{GRM}}$) and segment-based approaches (${\text{F}}_{\text{ROH}}$ and ${\text{F}}_{\text{HBD}}$). The ${\text{F}}_{\text{GRM}}$ for individual $i$ was taken as ${\mathbf{G}}_{ii}-1$ where $\mathbf{G}$ is the SNP derived GRM. The GRM was built according to VanRaden’s first method [22] and used a fixed allele frequency of 0.5 [23]. Numerous studies [23–25] have found an advantage in terms of higher correlations with pedigree or ROH inbreeding metrics when using a fixed allele frequency of 0.5 instead of calculating the allele frequencies of the base population. Genomic inbreeding using ROH (${\text{F}}_{\text{ROH}}$) was computed using the SNP1101 software [21] with a sliding window approach and a minimum number of SNPs in a window set to 20, a choice that was based on previous results from Liu et al. [26] and Forutan et al. [27], where higher (lower) thresholds for the minimum number of SNPs in a window were found to produce inbreeding coefficients that are underestimated (overestimated), the minimum base pair length was set at 1 Mbp, and the genotyping error rate was set at 0.01. The frequency of the unique ROH identified is in Additional file 1: Figure S1. In theory, ${\text{F}}_{\text{ROH}}$ represents the proportion of the autosomal genome that is made up of ROH segments and is given by ${\text{F}}_{{{\text{ROH}}}} = \sum\nolimits_{{i = 1}}^{n} {\frac{{L_{{ROH_{i} }} }}{{L_{{Genome}} }}}$; where ${L}_{{ROH}_{i}}$ is the length of the $i$th ROH and ${L}_{Genome}$ is the combined length of the autosomes covered by the markers. It is possible to identify the age of ROH inbreeding by looking at the length of the ROH, with shorter lengths generally indicating more ancient inbreeding, although the length of an ROH originating at a particular time (generation) can vary. In this respect, we created five inbreeding coefficients based on the proportion of the genome covered by ROH of length 1 to 2 Mb (${\text{F}}_{\text{ROH}1-2}$), 2 to 4 Mb (${\text{F}}_{\text{ROH}2-4}$), 4 to 8 Mb (${\text{F}}_{\text{ROH}4-8}$), 8 to 16 Mb (${\text{F}}_{\text{ROH}8-16}$), and 16 Mb or larger (${\text{F}}_{\text{ROH}16}$). The breakdown of the proportion of the genome covered by ROH of each of the length classes for 100 randomly sampled individuals is in Additional file 2: Figure S2. Other than using ROH length to identify inbreeding age classes, another segment-based approach that can be used is to model the probability that a marker is part of a homozygous-by-descent (HBD) segment using a hidden Markov model (HMM), where the length of an HBD segment is exponentially distributed and the probability to continue or stop an HBD segment between two markers separated by *d* morgans is ${e}^{-Rd}$, where $R$ is the rate of the exponential distribution [28]. This approach can identify classes of inbreeding age by the expected length of the HBD segment, where shorter segment lengths indicate more ancient ancestry. For this purpose, we used the RZooROH package [29] to model multiple HBD classes where the rates used followed a power of four series (4^n^; n = 1 to 4) to obtain partial inbreeding coefficients from approximately 2 (${\text{F}}_{\text{HBD}2}$), 8 (${\text{F}}_{\text{HBD}8}$), 32 (${\text{F}}_{\text{HBD}32}$), and 128 (${\text{F}}_{\text{HBD}128}$) generations ago. These estimates were non-cumulative and represent the proportion of inbreeding represented by HBD segment length classes that roughly correspond to a particular generation in the past.

### Impact of genomic selection on inbreeding and genetic diversity

**Fig. 1:**
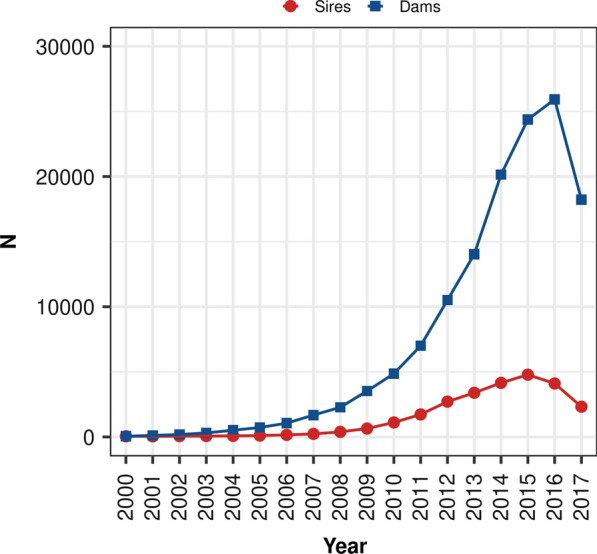
Number of genotyped sires and dams by year of birth. Each dot represents the number of genotyped sires (red) and dams (blue) born within a given year

To examine the impact of genomic selection on various genetic diversity metrics, genotyped animals with progeny were analyzed, specifically 26,149 sires and 135,548 dams born between 2000 and 2017. Animals that did not have recorded progeny were not considered in this analysis to correct any potential bias in the genetic merit of genotyped animals born before and after genomic selection. The number of genotyped sires and dams for each birth year considered is shown in Fig. 1. Sires and dams were further split into two birth year groups for subsequent analyses based on whether they were born before (PreGS; 2000–2009) or after the implementation of genomic testing and selection (PostGS; 2010–2017). In total, 1847 sires and 10,461 dams were born in the PreGS period and 24,302 sires and 125,087 dams were born in the PostGS period.

#### Rate of inbreeding, generation intervals, and effective population size

The yearly rate of inbreeding ($\Delta {\text{F}}_{\text{year}}$) using the pedigree ($\Delta {\text{F}}_{{\text{PED}}_{\text{year}}})$, marker-by-marker ($\Delta {\text{F}}_{{\text{GRM}}_{\text{year}}})$, and segment-based ($\Delta {\text{F}}_{{\text{ROH}}_{\text{year}}})$ inbreeding coefficients were calculated by regressing the inbreeding coefficient of the natural logarithm of (1-$\text{F}$) [7, 9] on the year of birth of the animal. The $\Delta {\text{F}}_{\text{year}}$ was obtained as the opposite of the slope of the regression and was calculated independently for PreGS and PostGS sires and dams. Animals with an inbreeding coefficient that was not within 3 standard deviations of the group mean were not considered for this analysis. To account for the disparity in number of animals between the groups, the regression was run on randomly sampled groups of 200 animals without replacement, with a total of 8, 117, 50, and 606 samplings done for PreGS and PostGS sires and dams, respectively. The mean of all the runs was taken as the estimate of $\Delta {\text{F}}_{\text{year}}$, and the standard deviation of all the runs divided by the square root of the number of runs was taken as the standard error of the estimate. Generation intervals were calculated for the traditional four paths of selection in each birth year group, these being the generation interval of sires of sires (${\text{L}}_{\text{SS}}$), sires of dams (${\text{L}}_{\text{SD}}$), dams of sires (${\text{L}}_{\text{DS}}$), and dams of dams (${\text{L}}_{\text{DD}}$). The generation intervals for all four paths of selection calculated at each birth year are in Additional file 3: Figure S3. The generation interval of the sires was taken as the average of ${\text{L}}_{\text{SS}}$ and ${\text{L}}_{\text{DS}}$ and the generation interval of the dams was taken as the average of ${\text{L}}_{\text{SD}}$ and ${\text{L}}_{\text{DD}}$. The effective population size (${N}_{e}$) within a sex and birth year group was calculated as $N_{e} = \frac{1}{{2{\text{L}}\Delta {\text{F}}_{{{\text{year}}}} }}$, where $\text{L}$ is the generation interval of either sires or dams of either the PreGS or PostGS group, and $\Delta {\text{F}}_{\text{year}}$ is the estimated yearly rate of inbreeding using one of the inbreeding coefficients. Similarly, a 95% confidence interval was constructed for the effective population size using $95\% {\text{CI}} = \frac{1}{{2{\text{L}}(\Delta {\text{F}}_{{{\text{year}}}} \pm 1.96{\text{~SE}}_{{\Delta {\text{F}}_{{{\text{year}}}} }} )}}$ , where ${\text{SE}}_{{\Delta \text{F}}_{\text{year}}}$ is the standard error of the estimated yearly rate of inbreeding.

#### Effective number of independently segregating chromosomal segments

The effective number of independently segregating chromosomal segments (${M}_{e}$) was calculated at every birth year for the sires and dams, independently, as follows:

$$M_{e} = ~\frac{1}{{Var\left( {G_{{ij}} - A_{{ij}} } \right)}},$$

where ${G}_{ij}$ and ${A}_{ij}$ are the relationships between the $i$th and $j$th animals based on the GRM and the numerator relationship matrix ($\mathbf{A}$), respectively. The GRM was scaled to the same base population as $\mathbf{A}$ using methods described in Wientjes et al. [30].

### Inbreeding depression for heifer pregnancy and growth traits

#### Traits

Records were obtained for 21,288 heifers for heifer pregnancy (HP), which is the binary phenotype of being either pregnant or open at the end of the breeding season. Records were also obtained for three different growth traits: birth weight (BiW), weaning weight (WW), and post-weaning gain (PWG) in males and females. Records for BiW, WW, and PWG were already adjusted for age of calf at time of measurement and records for BiW and WW were also adjusted for the age of dam. Only phenotypic records within three standard deviations of the mean were used. Summary statistics including number of animals, mean, median, standard deviation, and minimum and maximum values for HP, BiW, WW, and PWG, are in Table 1.

**Table 1:** Descriptive statistics (number of individuals (N), mean, median, standard deviation, minimum and maximum) for the fertility and growth traits analyzed

Trait^a^:	Group	N	Mean	Median	SD	Minimum	Maximum
HP		21,288	0.87^b^				
BiW (kg)	Males	224,098	36.56	36.73	4.00	24.49	49.89
	Females	151,801	34.32	34.47	3.91	22.68	47.17
WW (kg)	Males	222,759	310.90	311.60	41.04	185.00	443.10
	Females	148,792	277.60	278.00	34.75	170.50	390.90
PWG (kg)	Males	169,404	225.48	226.30	48.96	73.92	378.68
	Females	93,836	112.78	112.47	37.01	14.51	228.57

^a^*HP* heifer pregnancy, *BiW* birth weight, *WW* weaning weight, *PWG* post-weaning gain

^b^Represents the incidence of heifer pregnancy

#### Statistical analysis and modeling

The effect of a 1% increase of pedigree and genomic inbreeding coefficients relating to recent and ancient inbreeding on the phenotype of heifer pregnancy and growth traits was quantified using a linear mixed model approach. For growth traits, male and female growth was treated as distinct and analyzed with separate regression analysis in order to uncover potential differences between sexes for inbreeding depression. The model used to estimate the regression coefficients of ${\text{F}}_{\text{PED}}$, ${\text{F}}_{\text{GRM}}$, and ${\text{F}}_{\text{ROH}}$ was:

$${\mathbf{y}} = {\mathbf{Xb}} + \upbeta {\mathbf{F}} + {\mathbf{Za}} + \sum\limits_{{\text{i}}}^{{\text{n}}} {{\mathbf{Wr}}} + {\mathbf{e}},$$

where $\mathbf{y}$ is a vector of phenotypes for the investigated trait; $\mathbf{b}$ is a vector of fixed effects that included the contemporary group, age of dam, and heifer age when HP was modeled and contemporary group when BiW, WW, or PWG were modeled; $\mathbf{F}$ is a vector of inbreeding coefficients for ${\text{F}}_{\text{PED}}$, ${\text{F}}_{\text{GRM}}$, or ${\text{F}}_{\text{ROH}}$; ${\upbeta }$ is the linear regression coefficient for the regression coefficient; $\mathbf{a}$ is the vector of the random additive genetic effect, following ${\mathbf{a}}\sim N\left( {{\mathbf{0}},~{\mathbf{A}}\upsigma _{{{\text{add}}}}^{2} } \right)$, where $\mathbf{A}$ is the numerator relationship matrix; $\mathbf{r}$ is a vector of random effects that included the service sire (ss), following ${\mathbf{ss}}\sim N\left( {{\mathbf{0}},~{\mathbf{I}}\upsigma _{{{\text{ss}}}}^{2} } \right)$, where $\mathbf{I}$ is an identity matrix, when HP was modeled and the maternal permanent environment effect (mpe), following ${\mathbf{mpe}}\sim N\left( {{\mathbf{0}},~{\mathbf{I}}\upsigma _{{{\text{mpe}}}}^{2} } \right)$, when WW was modeled; $\mathbf{e}$ is the random residual, following ${\mathbf{e}}\sim N\left( {{\mathbf{0}},~{\mathbf{I}}\upsigma _{{\text{e}}}^{2} } \right)$; and $\mathbf{X}$, $\mathbf{Z}$, and $\mathbf{W}$ are the incidence matrices for the fixed and random effects. Variance components were fixed at values used in genetic evaluations by the AAA.

To estimate the effect of a 1% increase in recent and ancient pedigree and genomic inbreeding we arbitrarily grouped inbreeding classes based on whether they represent inbreeding acquired more recently or at a more distant generation, with the threshold for allocating the coefficients into one of the two classes being one of several that could be set. For pedigree inbreeding, we grouped inbreeding acquired 5 or fewer generations ago to create a recent pedigree inbreeding coefficient (${\text{F}}_{\text{PED}\_\text{REC}}={\text{F}}_{\text{PED}3}+{\text{F}}_{\text{PED}4-3}+{\text{F}}_{\text{PED}5-4}$) and inbreeding acquired 6 or more generations ago to create an ancient pedigree inbreeding coefficient (${\text{F}}_{\text{PED}\_\text{ANC}}={\text{F}}_{\text{PED}6-5}+{\text{F}}_{\text{PED}7-6}+{\text{F}}_{\text{PED}8-7}+{\text{F}}_{\text{PED}9+})$. To partition ROH inbreeding, we grouped inbreeding based on ROH longer than or equal to 8 Mb (approximately 6.25 generations ago or sooner) to create a recent ROH inbreeding coefficient (${\text{F}}_{\text{ROH}\_\text{REC}}={\text{F}}_{\text{ROH}16}+{\text{F}}_{\text{ROH}8-16}$) and inbreeding based on ROH segments shorter than 8 Mb (approximately from 6.25 to 50 generations ago) to create an ancient ROH inbreeding coefficient ${(\text{F}}_{\text{ROH}\_\text{ANC}}={\text{F}}_{\text{ROH}4-8}+{\text{F}}_{\text{ROH}2-4}+{\text{F}}_{\text{ROH}1-2})$. Similarly, for inbreeding based on HBD segments, we grouped the classes corresponding to inbreeding acquired approximately 2 and 8 generations ago to create a recent HBD inbreeding coefficient $({\text{F}}_{\text{HBD}\_\text{REC}}={\text{F}}_{\text{HBD}2}+{\text{F}}_{\text{HBD}8})$ and grouped the classes corresponding to inbreeding acquired approximately 32 and 128 generations ago to create an ancient HBD inbreeding class ${(\text{F}}_{\text{HBD}\_\text{ANC}}={\text{F}}_{\text{HBD}32}+{\text{F}}_{\text{HBD}128})$. The previous model was extended to fit the recent and ancient inbreeding coefficients of a certain class (example ${\text{F}}_{\text{PED}\_\text{REC}}$ and ${\text{F}}_{\text{PED}\_\text{ANC}}$) simultaneously.

Models where HP was the response variable were run using the THRGIBBS1F90 program (v.2.116) [31] and those in which the response variable was a growth trait were run using the GIBBS1F90 program (v.1.44) [32]. All analyses were run for 50,000 cycles with a burn-in of 10,000 samples and every 10th sample being stored, for a total of 4000 samples used for subsequent inference. Convergence was assessed by visual inspection of trace plots.

## Results and discussion

### Inbreeding measures

The mean inbreeding of the population was 5.92% (SD = 2.41%) for ${\text{F}}_{\text{PED}}$, 27.62% (SD = 2.52%) for ${\text{F}}_{\text{GRM}}$, and 16.24% (SD = 3.04%) for ${\text{F}}_{\text{ROH}}$. The mean ${\text{F}}_{\text{PED}}$ was much lower than that reported for line 1 Hereford cattle (29.2%) [24] but it was in line with estimates for different lineages of Nellore cattle (1 to 2%) [33]. The higher levels of pedigree inbreeding in line 1 Herefords than in American Angus are not surprising, as the former were managed as a closed herd for more than 85 years, which descends from only two bulls (paternal half-siblings) and 50 cows [34]. Using genomic inbreeding measures, the mean ${\text{F}}_{\text{GRM}}$ (30%) and ${\text{F}}_{\text{ROH}}$ (23%) of Herefords [24] were similar to the values calculated in our population, although ${\text{F}}_{\text{ROH}}$ was calculated using different ROH defining parameters, which limits the comparison between the two populations.

For the partial inbreeding coefficients, the mean inbreeding ranged from 0.10% (SD = 0.03%) for ${\text{F}}_{\text{PED}7-6}$ to 3.23% (SD = 2.39%) for ${\text{F}}_{\text{PED}3}$ according to pedigree-based coefficients, from 2.46% (SD = 0.40%) for ${\text{F}}_{\text{ROH}1-2}$ to 4.33% (SD = 1.00%) for ${\text{F}}_{\text{ROH}4-8}$ according to ROH segment-based coefficients, and from 0.00% (SD = 0.00%) for ${\text{F}}_{\text{HBD}32}$ to 25.26% (SD = 1.09%) for ${\text{F}}_{\text{HBD}128}$ according to HBD segment-based coefficients. Based on the partial inbreeding coefficients, the bulk of the pedigree inbreeding seems to have been accumulated relatively recently in the American Angus population. In contrast, the results for the partial inbreeding coefficients based on ROH indicate a more even distribution of ROH inbreeding accumulated recently and in more distant generations, and partial inbreeding coefficients based on HBD segments indicate a large part of inbreeding accumulated in a very distant past (around 128 generations ago) before the establishment of the modern American Angus breed indicating a possible historical bottleneck, and that a sizeable amount of inbreeding accumulated more recently, approximately eight generations ago. The distribution of pedigree and genomic inbreeding coefficients are in Additional file 4: Figure S4 and Additional file 5: Figure S5, respectively.

**Fig. 2:**
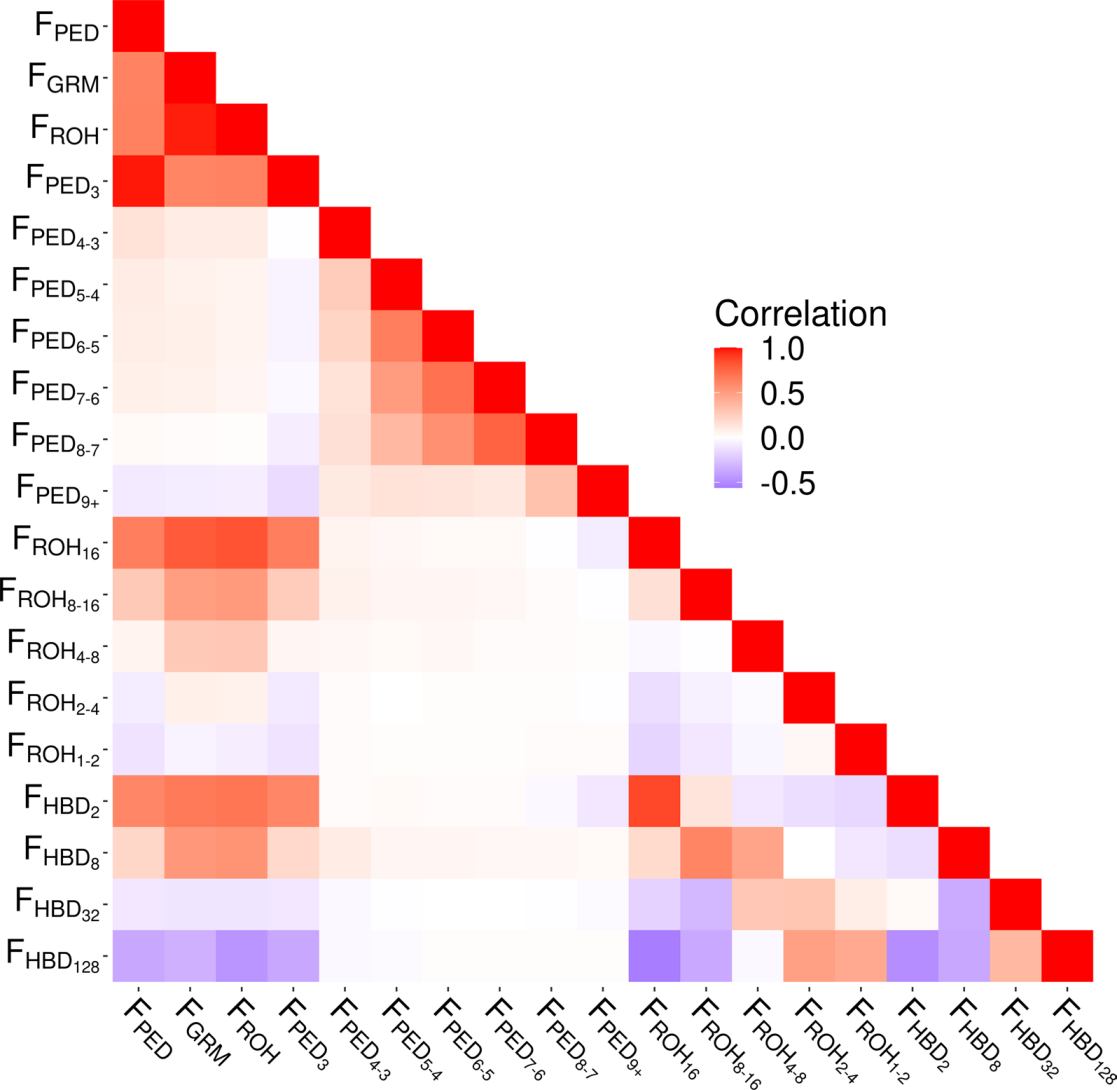
Pearson correlations between inbreeding measures. The plot shows the strength and direction of the correlation between inbreeding measures. Darker colors indicate a stronger correlation, while red and purple indicate a positive and negative correlation, respectively

Pearson’s correlation coefficients were calculated for all pairs of inbreeding coefficients and are shown in a heatmap in Fig. 2. The correlations of F_PED_ with ${\text{F}}_{\text{GRM}}$ and ${\text{F}}_{\text{ROH}}$ were high and positive (${\text{r}}_{{\text{F}}_{\text{PED}},{\text{F}}_{\text{GRM}}}=0.63$ and ${\text{r}}_{{\text{F}}_{\text{PED}},{\text{F}}_{\text{R}\text{O}\text{H}}}=0.64$), while the correlation of ${\text{F}}_{\text{G}\text{R}\text{M}}$ with ${\text{F}}_{\text{R}\text{O}\text{H}}$ was very high and positive (${\text{r}}_{{\text{F}}_{\text{G}\text{R}\text{M}},{\text{F}}_{\text{R}\text{O}\text{H}}}=0.97$). Similar to the results of this study, previously reported correlations between ${\text{F}}_{\text{PED}}$ and genomic inbreeding (${\text{F}}_{\text{G}\text{R}\text{M}}$ or ${\text{F}}_{\text{R}\text{O}\text{H}}$) and correlations between ${\text{F}}_{\text{G}\text{R}\text{M}}$ and ${\text{F}}_{\text{R}\text{O}\text{H}}$ range from moderate to high in beef and dairy cattle populations, with estimates of ${\text{r}}_{{\text{F}}_{\text{PED}},{\text{F}}_{\text{G}\text{R}\text{M}}}$ ranging from 0.43 to 0.64, estimates of ${\text{r}}_{{\text{F}}_{\text{PED}},{\text{F}}_{\text{R}\text{O}\text{H}}}$ ranging from 0.66 to 0.70, and estimates of ${\text{r}}_{{\text{F}}_{\text{G}\text{R}\text{M}},{\text{F}}_{\text{R}\text{O}\text{H}}}$ ranging from 0.81 to 0.92 [16, 24, 27].

The correlations of the classical inbreeding coefficient and the partial pedigree-based inbreeding coefficient ranged from low and negative (${\text{r}}_{{\text{F}}_{\text{PED}},{\text{F}}_{\text{PED}9+}}=-0.09$) to very high and positive (${\text{r}}_{{\text{F}}_{\text{PED}},{\text{F}}_{\text{PED}3}}=0.98$). Partial pedigree-based inbreeding coefficients, except for ${\text{F}}_{\text{PED}3}$, were found to share little to no correlation with genomic-based measures of inbreeding, with correlations ranging from − 0.08 to 0.08. ${\text{F}}_{\text{R}\text{O}\text{H}}$ and partial ROH segment-based inbreeding coefficients had correlations that ranged from low and negative (${\text{r}}_{{\text{F}}_{\text{R}\text{O}\text{H}},{\text{F}}_{\text{R}\text{O}\text{H}1-2}}=-0.07$) to high and positive (${\text{r}}_{{\text{F}}_{\text{R}\text{O}\text{H}},{\text{F}}_{\text{R}\text{O}\text{H}16}}=0.83$). We observed that partial inbreeding coefficients representing more recent inbreeding were more strongly correlated with each other than with those representing more ancient inbreeding, and vice versa, which was expected given that they represent a similar age of inbreeding accumulation.

### Genetic diversity in American Angus sires and dams

**Fig. 3:**
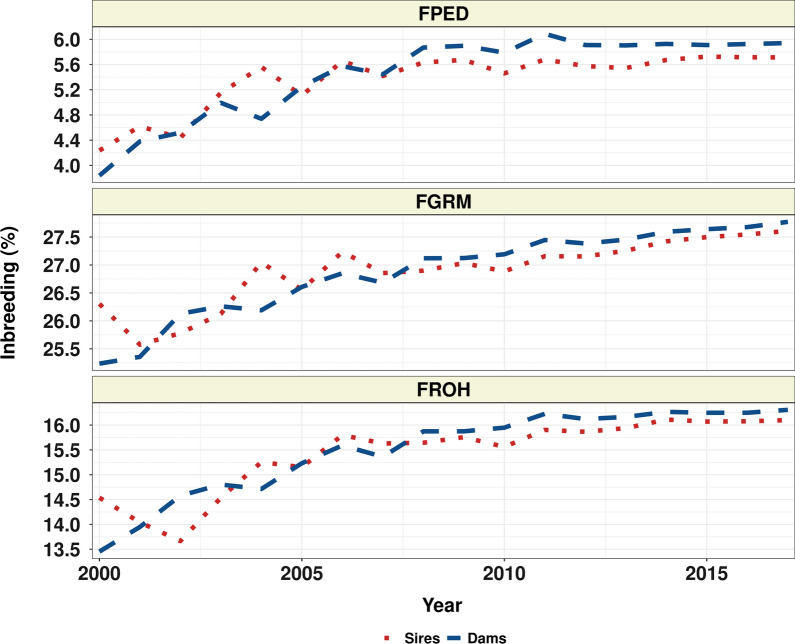
Mean pedigree and genomic inbreeding from 2000 to 2017. The plot shows the mean inbreeding in sires (red dots) and dams (blue lines) for each year from 2000 to 2017. The inbreeding measures shown include pedigree inbreeding (${\text{F}}_{\text{PED}}$), genomic GRM based inbreeding (${\text{F}}_{\text{G}\text{R}\text{M}}$), and ROH segment-based inbreeding (${\text{F}}_{\text{R}\text{O}\text{H}}$)

The average ${\text{F}}_{\text{PED}}$, ${\text{F}}_{\text{G}\text{R}\text{M}}$, and ${\text{F}}_{\text{R}\text{O}\text{H}}$, for sires and dams at each birth year, are shown in Fig. 3. There was an observable trend of increased average pedigree and genomic inbreeding from the first to last birth year considered, with the average ${\text{F}}_{\text{PED}}$ increasing by 34.67% and 54.69%, the average ${\text{F}}_{\text{G}\text{R}\text{M}}$ increasing by 4.98% and 10.02%, and the average ${\text{F}}_{\text{R}\text{O}\text{H}}$ increasing by 10.73% and 21.26% for sires and dams, respectively.

The results for the yearly change in inbreeding are in Table 2. In sires, we estimated a significant decrease in $\Delta {\text{F}}_{{\text{P}\text{E}\text{D}}_{\text{y}\text{e}\text{a}\text{r}}}$ from 0.14% in the PreGS period to 0.05% in the PostGS period; for genomic measures of inbreeding, we found a non-significant increase in $\Delta {\text{F}}_{{\text{G}\text{R}\text{M}}_{\text{y}\text{e}\text{a}\text{r}}}$ and a non-significant decrease in $\Delta {\text{F}}_{{\text{R}\text{O}\text{H}}_{\text{y}\text{e}\text{a}\text{r}}}$ from one period to another. In dams, we observed a significant decrease in $\Delta {\text{F}}_{\text{y}\text{e}\text{a}\text{r}}$ for all inbreeding coefficients considered, from 0.22 to 0.03% for $\Delta {\text{F}}_{{\text{P}\text{E}\text{D}}_{\text{y}\text{e}\text{a}\text{r}}}$, from 0.21 to 0.12% for $\Delta {\text{F}}_{{\text{G}\text{R}\text{M}}_{\text{y}\text{e}\text{a}\text{r}}}$, and from 0.23 to 0.07% for $\Delta {\text{F}}_{{\text{R}\text{O}\text{H}}_{\text{y}\text{e}\text{a}\text{r}}}$.

**Table 2:** Generation intervals (L), yearly rate of inbreeding ($\Delta {\text{F}}_{\text{y}\text{e}\text{a}\text{r}}$), and effective population size (${N}_{e}$)

Group	L (years)	$\Delta {\text{F}}_{{\text{P}\text{E}\text{D}}_{\text{y}\text{e}\text{a}\text{r}}}$(%)	${N}_{e\text{P}\text{E}\text{D}}$	$\Delta {\text{F}}_{{\text{G}\text{R}\text{M}}_{\text{y}\text{e}\text{a}\text{r}}}$(%)	${N}_{e\text{G}\text{R}\text{M}}$	$\Delta {\text{F}}_{{\text{R}\text{O}\text{H}}_{\text{y}\text{e}\text{a}\text{r}}}$(%)	${N}_{e\text{R}\text{O}\text{H}}$
Sires PreGS	5.15	0.14 (0.01)	71 (60, 88)	0.12 (0.03)	81 (57, 137)	0.14 (0.03)	70 (49, 124)
Sires PostGS	4.88	0.05 (0.01)	183 (149, 235)	0.15 (0.01)	65 (58, 74)	0.09 (0.01)	110 (91, 138)
Dams PreGS	5.43	0.22 (0.02)	41 (37, 48)	0.21 (0.02)	43 (37, 51)	0.23 (0.02)	40 (35, 47)
Dams PostGS	4.55	0.03 (0.003)	315 (263, 391)	0.12 (0.004)	75 (70, 80)	0.07 (0.004)	140 (124, 162)

Standard errors for $\Delta {\text{F}}_{\text{y}\text{e}\text{a}\text{r}}$ and 95% CI for ${N}_{e}$ are presented

*PreGS* pre genomic selection, *PostGS* post genomic selection, *PED* using pedigree, *GRM* using genomic relationship matrix, *ROH* using runs of homozygosity

These results are in stark contrast to those reported for dairy cattle. In North American dairy cattle, Makanjoula and colleagues [9] reported an increase for $\Delta {\text{F}}_{{\text{P}\text{E}\text{D}}_{\text{y}\text{e}\text{a}\text{r}}}$, $\Delta {\text{F}}_{{\text{G}\text{R}\text{M}}_{\text{y}\text{e}\text{a}\text{r}}}$, and $\Delta {\text{F}}_{{\text{R}\text{O}\text{H}}_{\text{y}\text{e}\text{a}\text{r}}}$ in Holsteins and Jerseys from 2000–2009 to 2010–2018. Similarly, Doekes et al. [7] and Doublet et al. [8] found a similar increase for $\Delta {\text{F}}_{{\text{P}\text{E}\text{D}}_{\text{y}\text{e}\text{a}\text{r}}}$ and $\Delta {\text{F}}_{{\text{R}\text{O}\text{H}}_{\text{y}\text{e}\text{a}\text{r}}}$ after the implementation of GS in Dutch-Flemish Holstein-Friesians and French Holstein-Friesians, respectively. However, not all dairy cattle breeds have been shaped by GS in the same way, e.g. $\Delta {\text{F}}_{{\text{R}\text{O}\text{H}}_{\text{y}\text{e}\text{a}\text{r}}}$ has not increased significantly in Normande and Montbeliarde in contrast to that in French Holstein-Friesians [8].

Generation intervals ($\text{L}$) were calculated for sires and dams at both periods and are in Table 2. We saw a decrease in the estimate for L in both sexes, with sire intervals decreasing from 5.15 to 4.88 years (a 5% decrease) and dam intervals decreasing from 5.43 to 4.55 years (a 16% decrease). This reduction in generation intervals after the introduction of genomic prediction was expected. In dairy cattle, a 25 to 50% reduction in generation intervals has been reported during the same period [3]. The larger decrease in generation intervals for dairy sires is likely due to the sex-limited nature of the dairy traits, which, before GS, required the bulls to enter progeny-testing to obtain accurate phenotypes for daughter performance, while in beef, growth traits can be measured on the selection candidates at an early age [2].

The estimates for yearly change in inbreeding and generation intervals were used to estimate the effective population sizes (${N}_{e}$) of the sire and dam populations at these periods and are in Table 2. In both sexes, we found a sharp increase in ${N}_{e\text{F}\text{P}\text{E}\text{D}}$ after 2010, from an ${N}_{e}$ size of 71 and 41 animals to that of 183 and 315 animals, in sires and dams, respectively. In sires, we found non-significant changes in ${N}_{e\text{G}\text{R}\text{M}}$ and ${N}_{e\text{R}\text{O}\text{H}}$ from the PreGS to PostGS periods. As for ${N}_{\text{e}\text{F}\text{P}\text{E}\text{D}}$, in dams, the estimated effective population sizes based on genomic inbreeding increased after 2010, ranging from a size increase of 32 animals for ${N}_{e\text{G}\text{R}\text{M}}$ to 100 animals for ${N}_{e\text{R}\text{O}\text{H}}$. These changes in ${N}_{e}$ size, which are seen most prominently in dams, are in the opposite direction of those seen in dairy cattle populations, where decreases in ${N}_{e}$ size have been observed after 2010 (roughly corresponding to the introduction of genomic testing in dairy) in North American and French populations [8, 9]. While the increases in ${N}_{e}$ sizes seen in the AAA dams indicate a considerable increase in genetic diversity after genomic selection, these results could be due to differences in the strategy used to select dams for genotyping in the different periods. We speculate that if selective genotyping took place in the PreGS period, this could have led to a pool of genotyped animals that do not adequately represent the true genetic diversity present during that period.

The effective number of independently segregating chromosome segments (${M}_{e}$) from 2000 to 2017 is in Additional file 6: Figure S6. In sires, ${M}_{e}$ ranged from 578 to 1230 segments with an average value of 905 segments in the PreGS period and ranged from 1066 to 1572 segments with an average value of 1305 segments in the PostGS period. Similarly, in dams, ${M}_{e}$ ranged from 623 to 1090 segments with an average value of 800 segments in the PreGS period and ranged from 765 to 1476 segments with an average value of 1162 segments in the PostGS period. The estimates of ${N}_{e}$ and ${M}_{e}$ and the observed decreases in the yearly rate of pedigree and genomic inbreeding suggest that genetic diversity in American Angus has increased in the last decade, coinciding with the implementation of genomic testing in this breed. While we tried to reduce sampling bias by only analyzing animals with offspring, there may be an effect of selective genotyping for PreGS animals that is difficult to disentangle and may be driving the results because they do not adequately represent the true genetic diversity present at that time. Another possibility is that mass genotyping has led to a larger pool of selection candidates that have, in turn, allowed a decrease in within-family selection and increased genetic diversity. However, any comparison of the present results with dairy populations relies on assuming a similar rate of adoption of GS strategies with similar effects on population structure in beef as in dairy breeds, which may not be completely accurate. Further studies should quantify the genetic diversity in other North American beef cattle breeds to see if similar trends are found.

### Pedigree and genomic inbreeding depression

In addition to the potential losses in genetic diversity, selection and mating practices that lead to the accumulation of inbreeding can cause a reduction in the mean fitness of individuals, or inbreeding depression. We quantified the magnitude and direction of the effect of pedigree and genomic inbreeding on heifer pregnancy (HP), and birth weight (BiW), weaning weight (WW), and post-weaning gain (PWG) in males and females. The effects of a 1% increase in ${\text{F}}_{\text{PED}}$, ${\text{F}}_{\text{G}\text{R}\text{M}}$, and ${\text{F}}_{\text{R}\text{O}\text{H}}$ in terms of phenotypic units and as a percentage of the trait mean are in Table 3. For HP, none of the three regression coefficients was significantly different from 0, with estimates for the decrease in pregnancy liability ranging from − 0.001 for ${\text{F}}_{\text{PED}}$ and ${\text{F}}_{\text{R}\text{O}\text{H}}$ to − 0.002 for ${\text{F}}_{\text{G}\text{R}\text{M}}$. Inbreeding depression for reproductive traits in beef and dairy cattle has been well documented, with pedigree or genomic inbreeding found to increase the age at first calving, calving intervals, and days open in Zebu and Alentejana cattle [13, 14]. In North American Holstein, inbreeding has been shown to increase the age at first service, the number of services and days open, and time from the first service to conception, and decrease conception rate [17, 25]. The majority of these traits share a moderate to high negative correlation with heifer or cow pregnancy ranging from − 0.41 to − 0.92 [35, 36].

**Table 3:** Inbreeding depression estimates for growth and heifer pregnancy expressed as change in the phenotype per 1% increase in inbreeding and as a percentage of the trait mean (% of $\stackrel{-}{x}$)

Trait	Group	${\text{F}}_{\text{PED}}$			${\text{F}}_{\text{G}\text{R}\text{M}}$			${\text{F}}_{\text{R}\text{O}\text{H}}$		
		Estimate	95% HPDI	% of $\stackrel{-}{x}$	Estimate	95% HPDI	% of $\stackrel{-}{x}$	Estimate	95% HPDI	% of $\stackrel{-}{x}$
HP^a^		− 0.001	(− 0.01, 0.01)		− 0.002	(− 0.01, 0.004)		− 0.002	(− 0.007, 0.004)	
BiW (kg)	Males	− 0.03	(− 0.04, − 0.03)	− 0.09	− 0.04	(− 0.05, − 0.03)	− 0.11	− 0.04	(− 0.04, − 0.03)	− 0.10
	Females	− 0.03	(− 0.04, − 0.02)	− 0.09	− 0.05	(− 0.05, − 0.04)	− 0.14	− 0.04	(− 0.05, − 0.03)	− 0.11
WW (kg)	Males	− 0.50	(− 0.55, − 0.44)	− 0.16	− 0.61	(− 0.66, − 0.57)	− 0.20	− 0.51	(− 0.55, − 0.48)	− 0.17
	Females	− 0.47	(− 0.55, − 0.40)	− 0.17	− 0.59	(− 0.65, − 0.53)	− 0.21	− 0.49	(− 0.54, − 0.44)	− 0.18
PWG (kg)	Males	− 0.64	(− 0.71, − 0.57)	− 0.28	− 0.72	(− 0.77, − 0.67)	− 0.32	− 0.59	(− 0.63, − 0.54)	− 0.26
	Females	− 0.34	(− 0.42, − 0.25)	− 0.30	− 0.42	(− 0.49, − 0.36)	− 0.37	− 0.35	(− 0.41, − 0.28)	− 0.31

*HP* heifer pregnancy, *BiW* birth weight, *WW* weaning weight, *PWG* post-weaning gain, ${F}_{PED}$ total pedigree inbreeding, ${F}_{GRM}$ genomic relationship matrix derived inbreeding, ${F}_{ROH}$ inbreeding based on runs of homozygosity

^a^Estimates for heifer pregnancy are given in the liability scale

When BiW was modeled, a 1% increase in ${\text{F}}_{\text{PED}}$ decreased BiW by 0.03 kg in both sexes, while increasing ${\text{F}}_{\text{G}\text{R}\text{M}}$ or ${\text{F}}_{\text{R}\text{O}\text{H}}$ decreased male birth weight by 0.04 kg for both genomic inbreeding measures and female birth weight by 0.05 kg and 0.04 kg for a 1% increase in ${\text{F}}_{\text{G}\text{R}\text{M}}$ and ${\text{F}}_{\text{R}\text{O}\text{H}}$_,_ respectively. The effect of a 1% increase in inbreeding on WW ranged from − 0.50 to − 0.61 kg in males and from − 0.47 to − 0.59 kg in females. The effect of a 1% increase in pedigree or genomic inbreeding on PWG was larger in males than females, with male PWG decreasing by − 0.64 kg for ${\text{F}}_{\text{PED}}$, − 0.72 kg for ${\text{F}}_{\text{G}\text{R}\text{M}}$, and − 0.59 kg for ${\text{F}}_{\text{R}\text{O}\text{H}}$, and female PWG decreasing by − 0.34 kg for ${\text{F}}_{\text{PED}}$, − 0.42 for ${\text{F}}_{\text{G}\text{R}\text{M}}$, and − 0.35 kg for ${\text{F}}_{\text{R}\text{O}\text{H}}$ in females. However, when depression is expressed in terms of the trait mean, a larger depressive effect is seen in females than males for most traits, and most notably for PWG when genomic inbreeding coefficients were used. In terms of percent change in the trait mean, the effect of increasing ${\text{F}}_{\text{PED}}$, ${\text{F}}_{\text{G}\text{R}\text{M}}$, and ${\text{F}}_{\text{R}\text{O}\text{H}}$ was more detrimental to PWG than any other trait. There is an observable trend of increasing growth depression across both sexes due to pedigree and genomic inbreeding load from birth to post-weaning. This result indicates that as the animal grows the reduction in the dam’s effect on offspring growth allows the inbreeding load of the animal to be increasingly reflected in its phenotypic value. In Hereford [24] and Angus cattle divergently selected for IGF-I concentrations [37], pedigree inbreeding has been found not to affect BiW while having a depressive effect on weaning weight and other post-weaning growth measures. Other studies in beef cattle have reported that a 1% increase in pedigree inbreeding depression decreases birth weight by 0.02 to 0.06 kg, decreases weaning weight by 0.19 to 0.44 kg, decreases adjusted 205-d weight by 0.25 kg, and decreases mature weight by 0.96 kg [13, 38, 39].

While studies on the effect of genomic inbreeding on growth are scarce, evidence of depression has been reported in Hereford and American Angus cattle. In line 1 Herefords, a 1% increase in ${\text{F}}_{\text{G}\text{R}\text{M}}$ has been associated with a 0.53 kg decrease in WW, which is similar to the effect found in the our study (− 0.61 kg in males and − 0.59 kg in females), however contrary to our findings, a depressive effect on BiW due to ${\text{F}}_{\text{G}\text{R}\text{M}}$ and on BiW and WW due to ${\text{F}}_{\text{R}\text{O}\text{H}}$ was not found [24]. In American Angus males, Garcia-Baccino et al. [40] also found a depressive effect of genomic inbreeding on growth with BiW decreasing by 0.05 kg, WW decreasing by 1.02 kg, and PWG decreasing by 1.07 kg per 1% increase in the proportion of homozygous SNPs (${\text{F}}_{\text{H}\text{O}\text{M}}$), a measure related to ${\text{F}}_{\text{G}\text{R}\text{M}}$ (using 0.5 as base allele frequency), albeit on a different scale.

To visualize the realized phenotypic depression in the Angus population, we calculated the projected loss in BiW, WW, and PWG, for the animals with low (5th percentile) and high (95th percentile) pedigree and genomic inbreeding, as well as the difference between the lowly and highly inbred animals. These results are in Table 4. The difference between the lowly and highly inbred animals ranged from 0.21 to 0.25 kg for male BiW, 0.20 to 0.31 kg for female BiW, 3.03 to 3.73 kg for male WW, 3.08 to 3.83 kg for female WW, 3.52 to 4.30 kg for male PWG, and 2.16 to 2.71 kg for female PWG. The biggest difference between lowly and highly inbred animals was found when inbreeding was measured using ${\text{F}}_{\text{G}\text{R}\text{M}}$, followed by ${\text{F}}_{\text{R}\text{O}\text{H}}$ for most traits.

**Table 4:** Projected phenotypic depression on growth of animals with low (5th percentile) and high (95th percentile) pedigree and genomic inbreeding

Trait	Group	${\text{F}}_{\text{PED}}$			${\text{F}}_{\text{G}\text{R}\text{M}}$			${\text{F}}_{\text{R}\text{O}\text{H}}$		
		Low	High	Difference	Low	High	Difference	Low	High	Difference
BiW (kg)	Males	− 0.12	− 0.33	0.21	− 0.15	− 0.40	0.25	− 0.13	− 0.34	0.22
	Females	− 0.11	− 0.31	0.20	− 0.17	− 0.47	0.31	− 0.14	− 0.39	0.25
WW (kg)	Males	− 1.75	− 4.78	3.03	− 2.15	− 5.88	3.73	− 1.81	− 4.94	3.13
	Females	− 1.66	− 4.74	3.08	− 2.07	− 5.90	3.83	− 1.74	− 4.96	3.22
PWG (kg)	Males	− 2.28	− 6.10	3.82	− 2.56	− 6.86	4.30	− 2.10	− 5.62	3.52
	Females	− 1.20	− 3.36	2.16	− 1.51	− 4.22	2.71	− 1.23	− 3.45	2.22

*BiW* birth weight, *WW* weaning weight, *PWG* post-weaning gain, ${F}_{PED}$ total pedigree inbreeding, ${F}_{GRM}$ genomic relationship matrix derived inbreeding, ${F}_{ROH}$ inbreeding based on runs of homozygosity

5th and 95th percentile inbreeding was calculated for each trait in males and females separately

Our results indicate that the current accumulation of pedigree and genomic inbreeding (see “Genetic diversity in American Angus sires and dams”) in the AAA population is detrimental to animal growth performance. However, current trends in genetic gain for weaning and yearling weight for AAA registered animals (http://www.angus.org/nce/genetictrends.aspx) show continuous improvement for these traits. These gains indicate a larger effect of selected regions of the genome for growth genetic gain than the detrimental impact caused by the correlated increase in inbreeding. Therefore, careful monitoring of genetic diversity and inbreeding load in the AAA population is essential to prevent the reversal of this trend.

### Effects of recent and ancient inbreeding

**Fig. 4:**
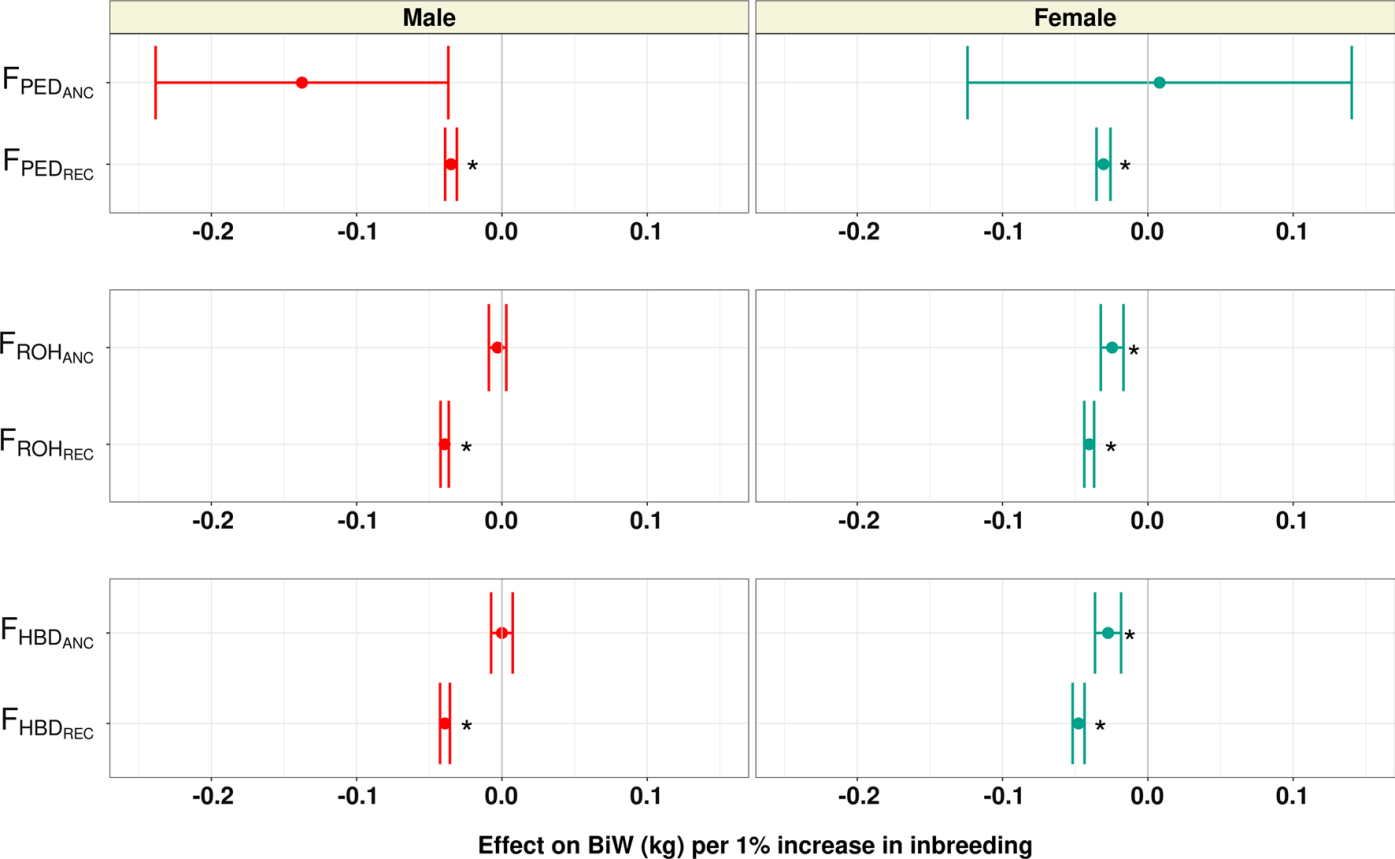
Effect of a 1% increase in recent and ancient pedigree and genomic inbreeding on birth weight (BiW; kg). An interval of the estimate $\pm$ SE is shown

**Fig. 5:**
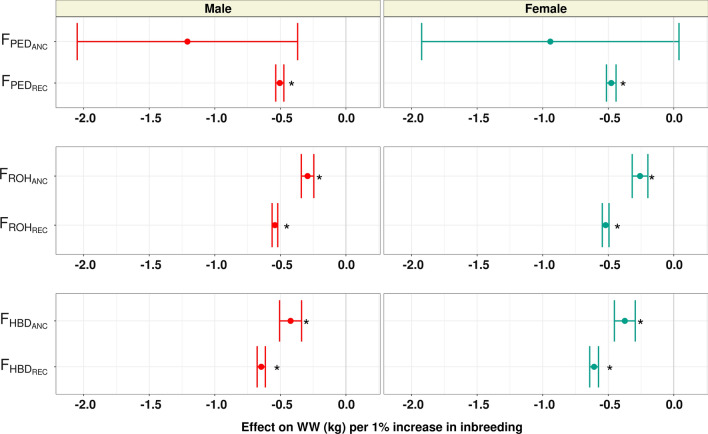
Effect of a 1% increase in recent and ancient pedigree and genomic inbreeding on weaning weight (WW; kg). An interval of the estimate $\pm$ SE is shown

**Fig. 6:**
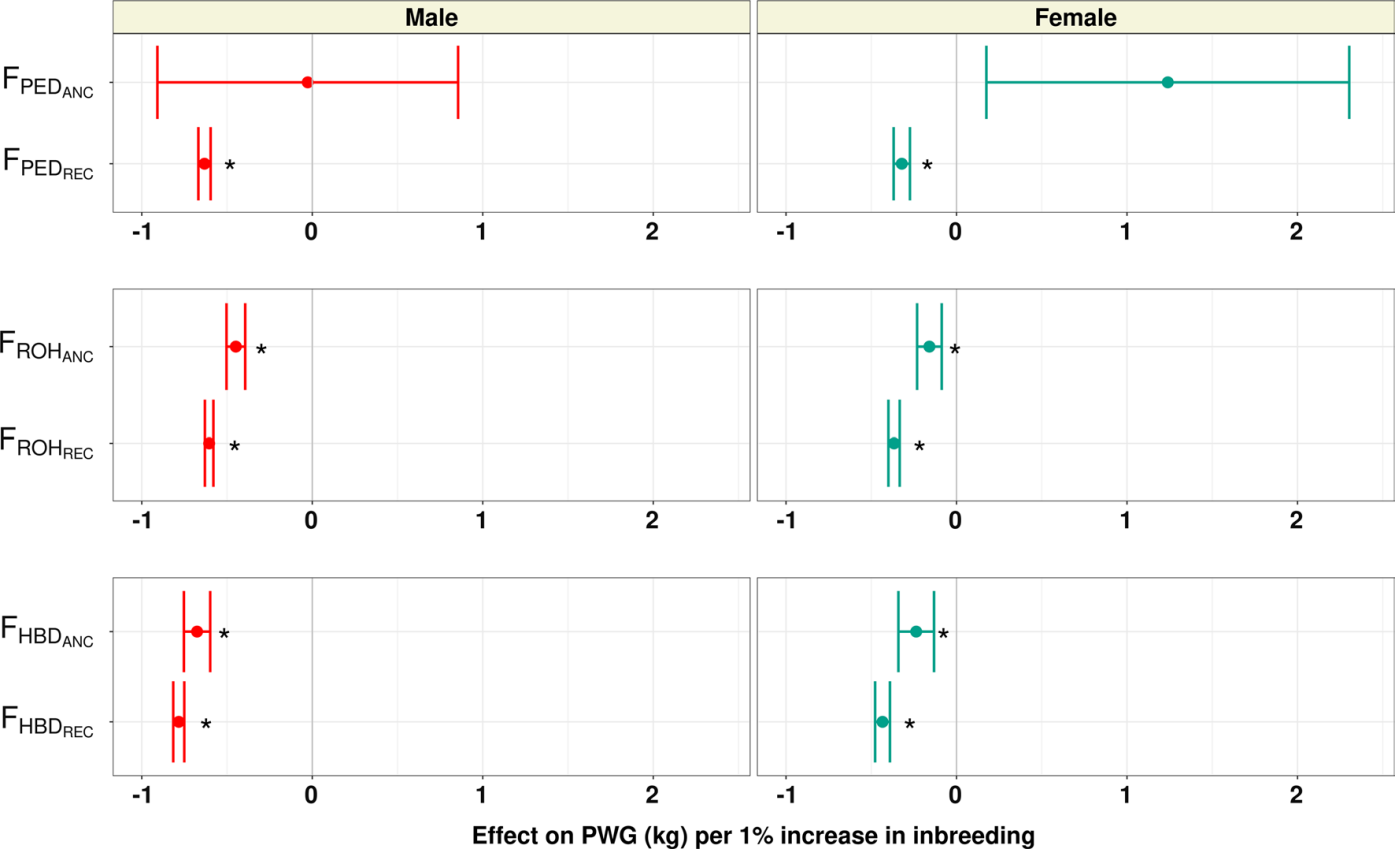
Effect of a 1% increase in recent and ancient pedigree and genomic inbreeding on post-weaning gain (PWG; kg). An interval of the estimate $\pm$ SE is shown

In this study, we determined the effect of the accumulation of recent and ancient pedigree and genomic inbreeding. The effects of a 1% increase in recent (${\text{F}}_{\text{PED}\_\text{R}\text{E}\text{C}}$, ${\text{F}}_{\text{R}\text{O}\text{H}\_\text{R}\text{E}\text{C}}$, and ${\text{F}}_{\text{H}\text{B}\text{D}\_\text{R}\text{E}\text{C}}$) and ancient (${\text{F}}_{\text{PED}\_\text{A}\text{N}\text{C}}$, ${\text{F}}_{\text{R}\text{O}\text{H}\_\text{A}\text{N}\text{C}}$, and ${\text{F}}_{\text{H}\text{B}\text{D}\_\text{A}\text{N}\text{C}}$) inbreeding for BiW, WW, and PWG, are shown in Figs. 4, 5, and 6, respectively, and for HP these results are in Additional file 7: Figure S7.

For HP, similar to the results for ${\text{F}}_{\text{PED}}$, ${\text{F}}_{\text{G}\text{R}\text{M}}$, and ${\text{F}}_{\text{R}\text{O}\text{H}}$ inbreeding depression, we found no significant effect for any of the recent or ancient inbreeding coefficients studied. In dairy cattle, multiple studies have found either no effect of recent and ancient inbreeding on fertility traits [16] or a much less pronounced effect than depression for production traits [17, 41]. In the cases where these studies found inbreeding depression, recent pedigree and genomic inbreeding were more detrimental to fertility than ancient inbreeding, and affected the age at first service, number of services, intervals from the first service to conception and from conception to the first service, non-return rate, and pregnancy rate in cows and heifers [17, 41].

As expected, we found a generally more extensive and more detrimental effect of recent pedigree or recent genomic inbreeding than ancient inbreeding for the growth traits analyzed. While ${\text{F}}_{\text{PED}-\text{R}\text{E}\text{C}}$ had a significant effect for all measures of growth and in both sexes, all the estimates of the effect of ${\text{F}}_{\text{PED}\_\text{A}\text{N}\text{C}}$ had substantial standard errors and were non-significant. For the most part, a 1% increase in ${\text{F}}_{\text{PED}\_\text{R}\text{E}\text{C}}$ had an effect on growth that is comparable in magnitude to the same increase in overall inbreeding (${\text{F}}_{\text{PED}}$); with BiW decreasing by 0.04 and 0.03 kg, WW decreasing by 0.5 and 0.48 kg, and PWG decreasing by 0.62 and 0.32 kg, in males and females, respectively. The larger unfavorable effect of recent pedigree inbreeding over ancient inbreeding was previously reported in dairy cattle. In both Dutch and North American Holsteins, recent pedigree inbreeding was found to be detrimental to milk, fat, and protein yield, while more ancient inbreeding did not affect these traits [16, 17]. In addition to the coefficients studied, other genealogical measures of ancestral inbreeding based on purging have been developed by Ballou [42] and Kalinowski [43] and have been found to have moderate to high correlations with recent pedigree inbreeding [44]. Further studies should be done using purging-based measures to validate the results found for growth depression using recent and ancient inbreeding based on generations.

Recent and ancient ROH inbreeding was significantly associated with a phenotypic decrease for all traits, except for male BiW for which ${\text{F}}_{\text{R}\text{O}\text{H}\_\text{A}\text{N}\text{C}}$ had no effect. For birth weight, a 1% increase in ${\text{F}}_{\text{R}\text{O}\text{H}\_\text{R}\text{E}\text{C}}$ decreased birth weight by 0.04 kg in males and females, while a 1% increase in ${\text{F}}_{\text{R}\text{O}\text{H}\_\text{A}\text{N}\text{C}}$ decreased female birth weight by 0.03 kg. For both weaning weight and post-weaning gain, an increase in ${\text{F}}_{\text{R}\text{O}\text{H}\_\text{R}\text{E}\text{C}}$ was noticeably more detrimental to weight gain than ${\text{F}}_{\text{R}\text{O}\text{H}\_\text{A}\text{N}\text{C}}$. A 1% increase in ${\text{F}}_{\text{R}\text{O}\text{H}\_\text{R}\text{E}\text{C}}$ decreased WW by 0.54 kg in males and 0.52 kg in females and decreased PWG by 0.60 kg in males and 0.37 kg in females. Meanwhile, the same increase in ${\text{F}}_{\text{R}\text{O}\text{H}\_\text{A}\text{N}\text{C}}$ decreased WW by 0.29 kg in males and 0.26 kg in females and decreased PWG by 0.45 kg in males and 0.16 kg in females.

Recent HBD inbreeding had a significant effect on all traits studied in both sexes, with a 1% increase in inbreeding decreasing BiW by 0.04 kg in males and 0.05 kg in females, decreasing WW by 0.65 kg in males and 0.61 kg in females, and decreasing PWG by 0.78 kg in males and 0.43 kg in females. The estimates for the effect of ${\text{F}}_{\text{H}\text{B}\text{D}-\text{A}\text{N}\text{C}}$ did not converge for WW in both sexes and PWG in males. Increased ${\text{F}}_{\text{H}\text{B}\text{D}-\text{A}\text{N}\text{C}}$ was associated with a decrease of 0.03 kg for BiW and 0.24 kg for PWG in females but did not affect male BiW. In dairy cattle, recent genomic inbreeding (ROH and HBD inbreeding) has mainly been more detrimental to performance than ancient inbreeding [16, 17, 41]. The results of our analysis confirm the largely unfavorable effects of recently accumulated inbreeding on animal performance. In addition, our results suggest that partitioning genomic inbreeding by age can help monitor inbreeding accumulation and better understand the relationship between total inbreeding load and expected inbreeding depression.

## Conclusions

In the present study, we measured the changes in the genetic diversity of American Angus cattle by estimating yearly and generational inbreeding rate, generation intervals, effective population sizes, and the effective number of independently segregating chromosome segments in sires and dams born between 2000 and 2017. Our results indicate that across all the metrics used, genetic diversity has been conserved in American Angus and, in some cases, has even increased after the implementation of genomic selection. Although we found evidence of a reduction in pedigree and genomic inbreeding accumulation rates, the average inbreeding in the population has continued to increase and should be monitored. In addition, we looked at pedigree and genomic inbreeding depression for fertility and growth. We found that increased genomic inbreeding had a larger effect on growth than pedigree-based measures, depressive effects for growth increased from birth to post-weaning, and that recent pedigree and genomic inbreeding was more harmful to growth than inbreeding accumulated during a more ancient period, confirming results that have been reported in similar studies on beef and dairy cattle. We did not find evidence of a depressive effect of pedigree or genomic inbreeding on heifer pregnancy, which is in line with other studies that have seen more significant depression for production traits than fertility in cattle.

## Supplementary Information

**Additional file 1: Figure S1.** Frequency of ROH across the genome (*Bos taurus* chromosome (BTA)1 – BTA29). Number of animals sharing a unique ROH by chromosome position.

**Additional file 2: Figure S2.** Proportion of genome covered by ROH of different lengths. Proportion of the genome covered by ROH of lengths 1–2, 2–4, 4–8, 8–16, and larger than 16 Mb for 100 randomly sampled individuals in the population.

**Additional file 3: Figure S3.** Generation intervals for the four paths of selection (2000–2017). Generation intervals calculated for the sires of sires’, dams of sires’, sires of dams’, and dams of dams’ paths of selection calculated for every year of birth from 2000 to 2017.

**Additional file 4: Figure S4.** Distribution of pedigree inbreeding. Distribution of total pedigree inbreeding and partial pedigree inbreeding.

**Additional file 5: Figure S5.** Distribution of genomic inbreeding. Distribution of genomic inbreeding based on the diagonal of the genomic relationship matrix, based on ROH and partial ROH inbreeding, and model-based HBD segment inbreeding coefficients.

**Additional file 6: Figure S6.** Effective number of independently segregating chromosome segments (2000–2017). Effective number of independently segregating chromosome segments calculated for sires and dams from 2000 to 2017.

**Additional file 7: Figure S7.** Effect of a 1% increase in recent and ancient inbreeding on heifer pregnancy. Estimates for the effect of a 1% increase in recent and ancient pedigree and genomic inbreeding.

## Data Availability

Not applicable.

## References

[1] Nejati-Javaremi A, Smith C, Gibson JP (1997). Effect of total allelic relationship on accuracy of evaluation and response to selection. J Anim Sci.

[2] Meuwissen T, Hayes B, Goddard M (2016). Genomic selection: A paradigm shift in animal breeding. Anim Front.

[3] García-Ruiz A, Cole JB, VanRaden PM, Wiggans GR, Ruiz-López FJ, Van Tassell CP (2016). Changes in genetic selection differentials and generation intervals in US Holstein dairy cattle as a result of genomic selection. Proc Natl Acad Sci USA.

[4] Wiggans GR, Cole JB, Hubbard SM, Sonstegard TS (2017). Genomic selection in Dairy Cattle: the USDA experience. Annu Rev Anim Biosci.

[5] Schaeffer LR (2006). Strategy for applying genome-wide selection in dairy cattle. J Anim Breed Genet.

[6] Daetwyler HD, Villanueva B, Bijma P, Woolliams JA (2007). Inbreeding in genome-wide selection. J Anim Breed Genet.

[7] Doekes HP, Veerkamp RF, Bijma P, Hiemstra SJ, Windig JJ (2018). Trends in genome-wide and region-specific genetic diversity in the Dutch-Flemish Holstein-Friesian breeding program from 1986 to 2015. Genet Sel Evol.

[8] Doublet AC, Croiseau P, Fritz S, Michenet A, Hozé C, Danchin-Burge C (2019). The impact of genomic selection on genetic diversity and genetic gain in three French dairy cattle breeds. Genet Sel Evol.

[9] Makanjuola BO, Miglior F, Abdalla EA, Maltecca C, Schenkel FS, Baes CF (2020). Effect of genomic selection on rate of inbreeding and coancestry and effective population size of Holstein and Jersey cattle populations. J Dairy Sci.

[10] Meuwissen THE, Sonesson AK, Gebregiwergis G, Woolliams JA (2020). Management of genetic diversity in the era of genomics. Front Genet.

[11] Falconer DS, Mackay TFC (1996). Introduction to quantitative genetics.

[12] Charlesworth D, Willis JH (2009). The genetics of inbreeding depression. Nat Rev Genet.

[13] Carolino N, Gama LT (2008). Inbreeding depression on beef cattle traits: Estimates, linearity of effects and heterogeneity among sire-families. Genet Sel Evol.

[14] Pereira RJ, Santana ML, Ayres DR, Bignardi AB, Menezes GRO, Silva LOC (2016). Inbreeding depression in Zebu cattle traits. J Anim Breed Genet.

[15] Reverter A, Porto-Neto LR, Fortes MRS, Kasarapu P, De Cara MAR, Burrow HM (2017). Genomic inbreeding depression for climatic adaptation of tropical beef cattle. J Anim Sci.

[16] Doekes HP, Veerkamp RF, Bijma P, de Jong G, Hiemstra SJ, Windig JJ (2019). Inbreeding depression due to recent and ancient inbreeding in Dutch Holstein-Friesian dairy cattle. Genet Sel Evol.

[17] Makanjuola BO, Maltecca C, Miglior F, Schenkel FS, Baes CF (2020). Effect of recent and ancient inbreeding on production and fertility traits in Canadian Holsteins. BMC Genomics.

[18] Wellmann R (2019). Optimum contribution selection for animal breeding and conservation: the R package optiSel. BMC Bioinformatics.

[19] MacCluer JW, Boyce AJ, Dyke B, Weitkamp LR, Pfenning DW, Parsons CJ (1983). Inbreeding and pedigree structure in Standardbred horses. J Hered.

[20] Sargolzaei M, Chesnais JP, Schenkel FS (2014). A new approach for efficient genotype imputation using information from relatives. BMC Genomics.

[21] Sargolzaei M (2014). SNP1101 User’s Guide. Version 1.

[22] VanRaden PM (2008). Efficient methods to compute genomic predictions. J Dairy Sci.

[23] VanRaden PM, Olson KM, Wiggans GR, Cole JB, Tooker ME (2011). Genomic inbreeding and relationships among Holsteins, Jerseys, and Brown Swiss. J Dairy Sci.

[24] Sumreddee P, Toghiani S, Hay EH, Roberts A, Agrrey SE, Rekaya R (2019). Inbreeding depression in line 1 Hereford cattle population using pedigree and genomic information. J Anim Sci.

[25] Bjelland DW, Weigel KA, Vukasinovic N, Nkrumah JD (2013). Evaluation of inbreeding depression in Holstein cattle using whole-genome SNP markers and alternative measures of genomic inbreeding. J Dairy Sci.

[26] Liu H, Sørensen AC, Meuwissen THE, Berg P (2014). Allele frequency changes due to hitch-hiking in genomic selection programs. Genet Sel Evol.

[27] Forutan M, Ansari Mahyari S, Baes C, Melzer N, Schenkel FS, Sargolzaei M (2018). Inbreeding and runs of homozygosity before and after genomic selection in North American Holstein cattle. BMC Genomics.

[28] Druet T, Gautier M (2017). A model-based approach to characterize individual inbreeding at both global and local genomic scales. Mol Ecol.

[29] Bertrand AR, Kadri NK, Flori L, Gautier M, Druet T (2019). RZooRoH: An R package to characterize individual genomic autozygosity and identify homozygous-by-descent segments. Methods Ecol Evol.

[30] Wientjes YCJ, Bijma P, Veerkamp RF, Calus MPL (2016). An equation to predict the accuracy of genomic values by combining data from multiple traits, populations, or environments. Genetics.

[31] Tsuruta S, Misztal I. THRGIBBS1F90 for estimation of variance components with threshold and linear models. In: Proceedings of the 8th world congress on genetics applied to livestock production: 13–18 August 2006; Minas Gerais; 2006.

[32] Misztal I, Tsuruta S, Strabel T, Auvray B, Druet T, Lee DH, et al. BLUPF90 and related programs (BGF90). In: Proceedings of the 7th world congress on genetics applied to livestock production: 19–23 August 2002; Montpellier; 2002. p. 743–4.

[33] Peripolli E, Metzger J, De Lemos MVA, Stafuzza NB, Kluska S, Olivieri BF (2018). Autozygosity islands and ROH patterns in Nellore lineages: Evidence of selection for functionally important traits. BMC Genomics.

[34] MacNeil MD (2009). Invited review: research contributions from seventy-five years of breeding Line 1 Hereford cattle at Miles City, Montana. J Anim Sci.

[35] Berry DP, Wall E, Pryce JE (2014). Genetics and genomics of reproductive performance in dairy and beef cattle. Animal.

[36] Eler JP, Bignardi AB, Ferraz JBS, Santana JL (2014). Genetic relationships among traits related to reproduction and growth of Nelore females. Theriogenology.

[37] Davis ME, Simmen RCM (2010). Estimates of inbreeding depression for serum insulin-like growth factor I concentrations, body weights, and body weight gains in Angus beef cattle divergently selected for serum insulin-like growth factor I concentration. J Anim Sci.

[38] Pariacote F, Van Vleck LD, MacNeil MD (1998). Effects of inbreeding and heterozygosity on preweaning traits in a closed population of Herefords under selection. J Anim Sci.

[39] Carrillo JA, Siewerdt F (2010). Consequences of long-term inbreeding accumulation on preweaning traits in a closed nucleus Angus herd. J Anim Sci.

[40] Garcia-Baccino CA, Lourenco DAL, Miller S, Cantet RJC, Vitezica ZG (2020). Estimating dominance genetic variances for growth traits in American Angus males using genomic models. J Anim Sci.

[41] Maltecca C, Tiezzi F, Cole JB, Baes C (2020). Symposium review: Exploiting homozygosity in the era of genomics: selection, inbreeding, and mating programs. J Dairy Sci.

[42] Ballou JD (1997). Ancestral inbreeding only minimally affects inbreeding depression in mammalian populations. J Hered.

[43] Kalinowski ST, Hedrick PW, Miller PS (2000). Inbreeding depression in the Speke’s gazelle captive breeding program. Conserv Biol.

[44] Rodríguez-Ramilo ST, Reverter A, Sánchez JP, Fernández J, Velasco-Galilea M, González O (2020). Networks of inbreeding coefficients in a selected population of rabbits. J Anim Breed Genet.

