# Supplementary figures and images for: Trends in genetic diversity and the effect of inbreeding in American Angus cattle under genomic selection
Source: Genet Sel Evol. 2021 Jun 16;53:50. doi: 10.1186/s12711-021-00644-z (PMC8207663; doi:10.1186/s12711-021-00644-z)

**
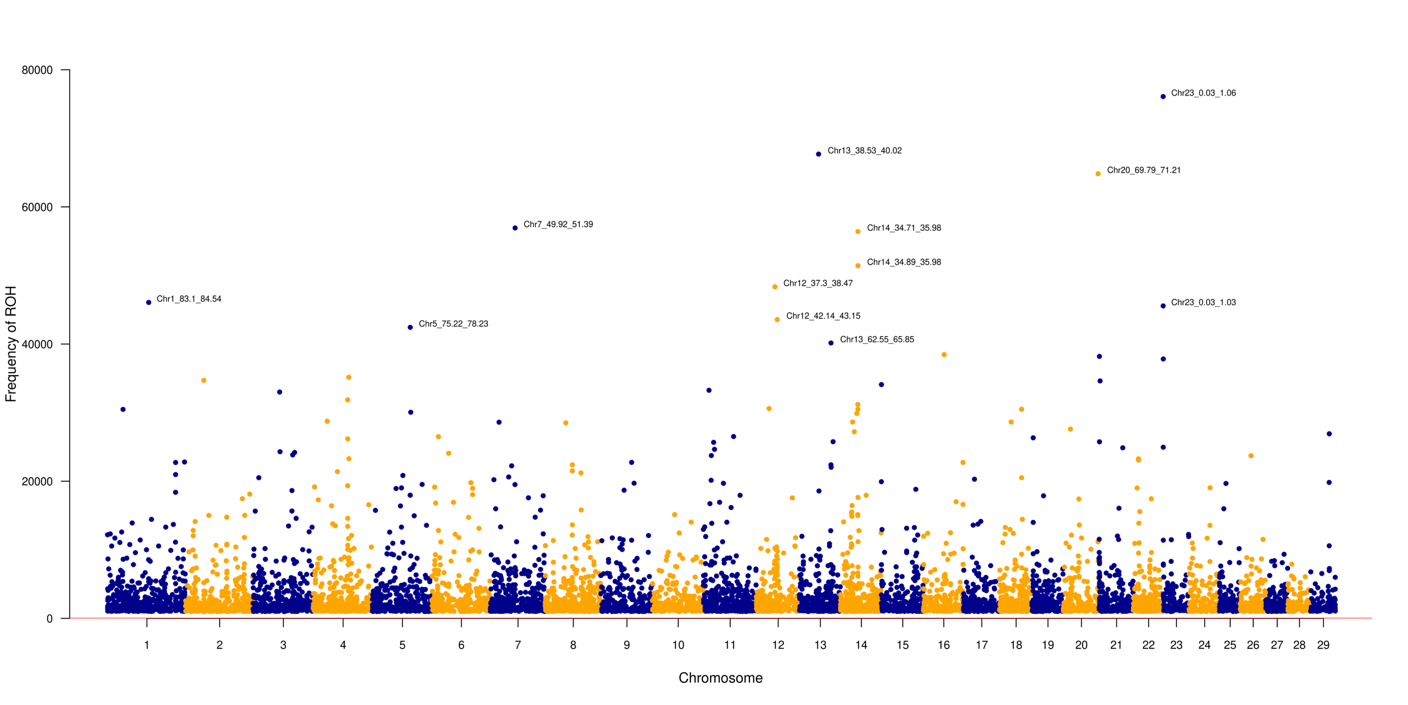
**

Supplement: Supplementary file 1 — Additional file 1: Figure S1. Frequency of ROH across the genome (Bos taurus chromosome (BTA)1 – BTA29). Number of animals sharing a unique ROH by chromosome position. [file 12711_2021_644_MOESM1_ESM.docx]

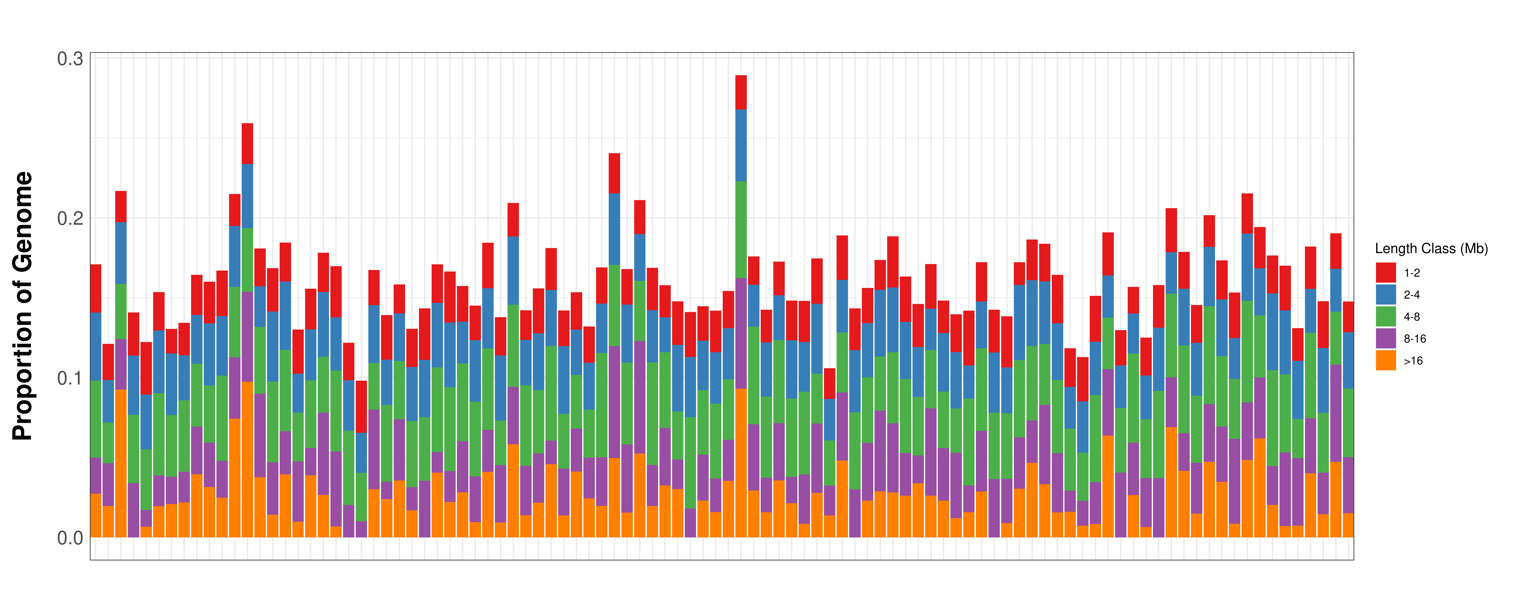

Supplement: Supplementary file 2 — Additional file 2: Figure S2. Proportion of genome covered by ROH of different lengths. Proportion of the genome covered by ROH of lengths 1–2, 2–4, 4–8, 8–16, and larger than 16 Mb for 100 randomly sampled individuals in the population. [file 12711_2021_644_MOESM2_ESM.docx]

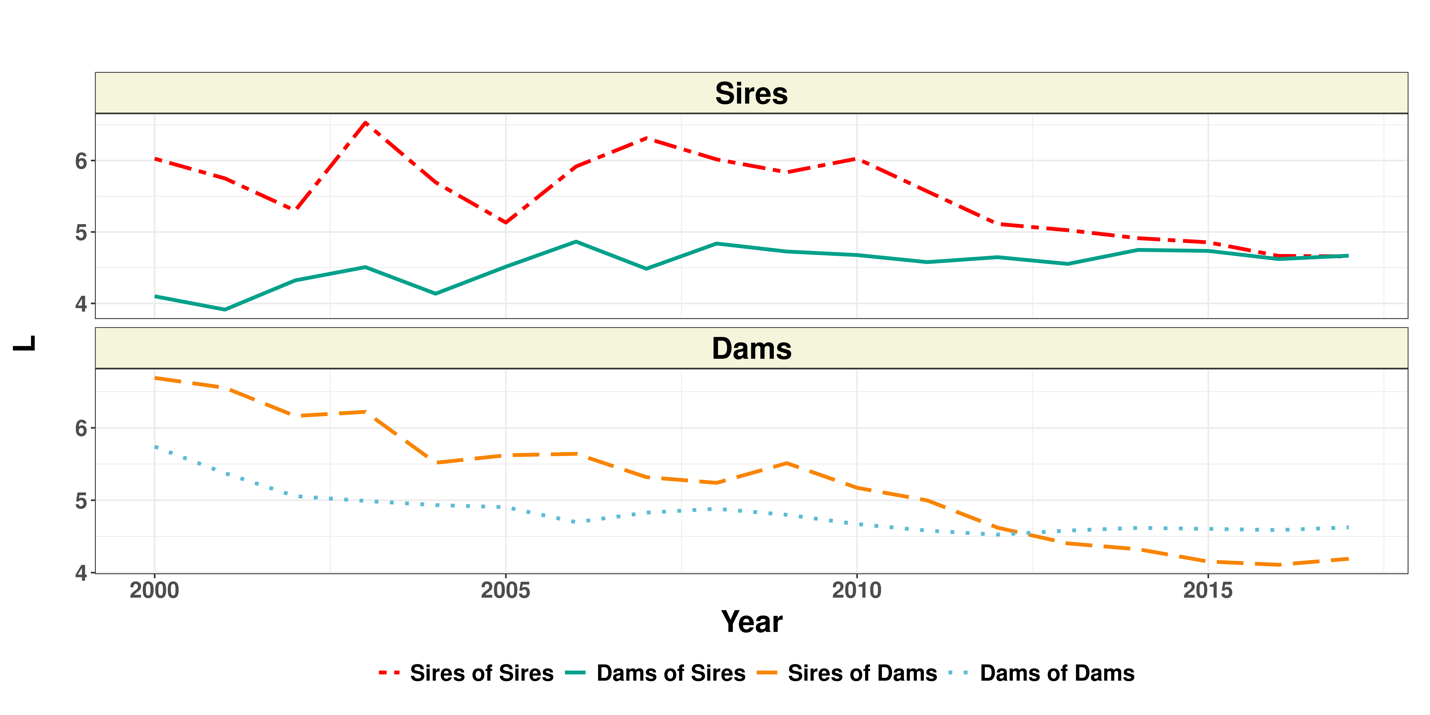

Supplement: Supplementary file 3 — Additional file 3: Figure S3. Generation intervals for the four paths of selection (2000–2017). Generation intervals calculated for the sires of sires’, dams of sires’, sires of dams’, and dams of dams’ paths of selection calculated for every year of birth from 2000 to 2017. [file 12711_2021_644_MOESM3_ESM.docx]

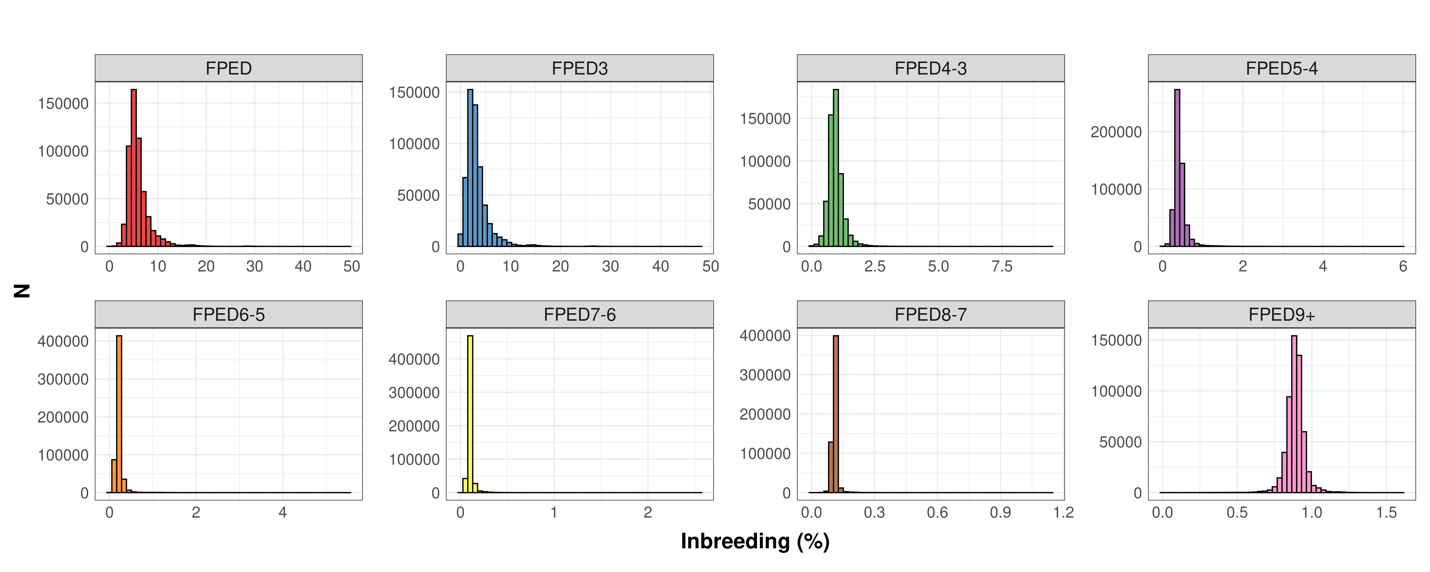

Supplement: Supplementary file 4 — Additional file 4: Figure S4. Distribution of pedigree inbreeding. Distribution of total pedigree inbreeding and partial pedigree inbreeding. [file 12711_2021_644_MOESM4_ESM.docx]

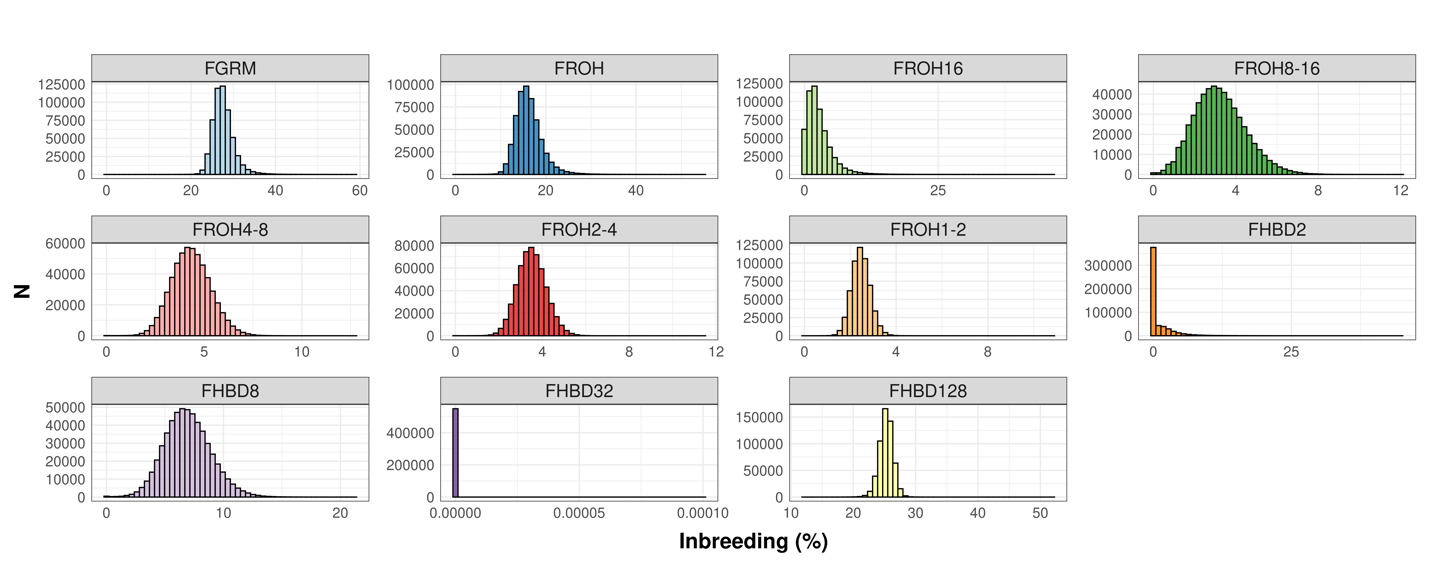

Supplement: Supplementary file 5 — Additional file 5: Figure S5. Distribution of genomic inbreeding. Distribution of genomic inbreeding based on the diagonal of the genomic relationship matrix, based on ROH and partial ROH inbreeding, and model-based HBD segment inbreeding coefficients. [file 12711_2021_644_MOESM5_ESM.docx]

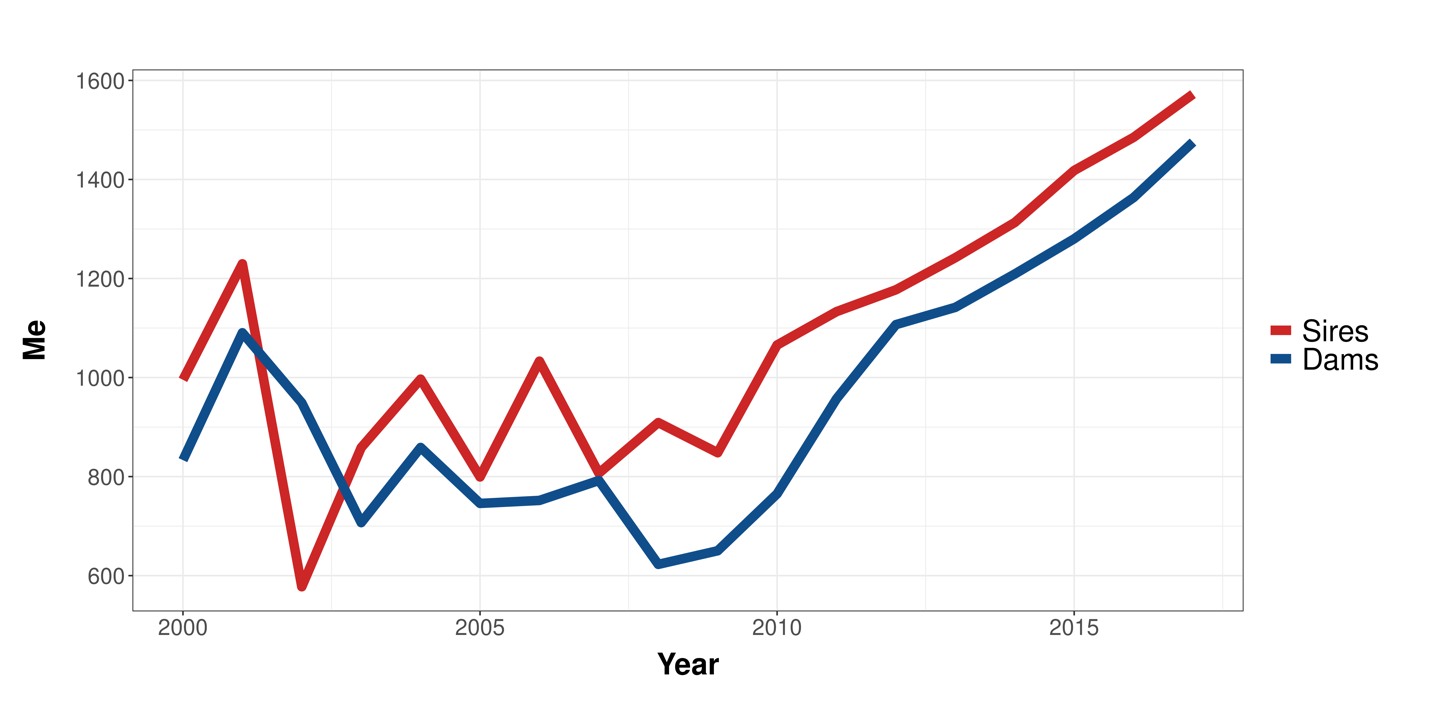

Supplement: Supplementary file 6 — Additional file 6: Figure S6. Effective number of independently segregating chromosome segments (2000–2017). Effective number of independently segregating chromosome segments calculated for sires and dams from 2000 to 2017. [file 12711_2021_644_MOESM6_ESM.docx]

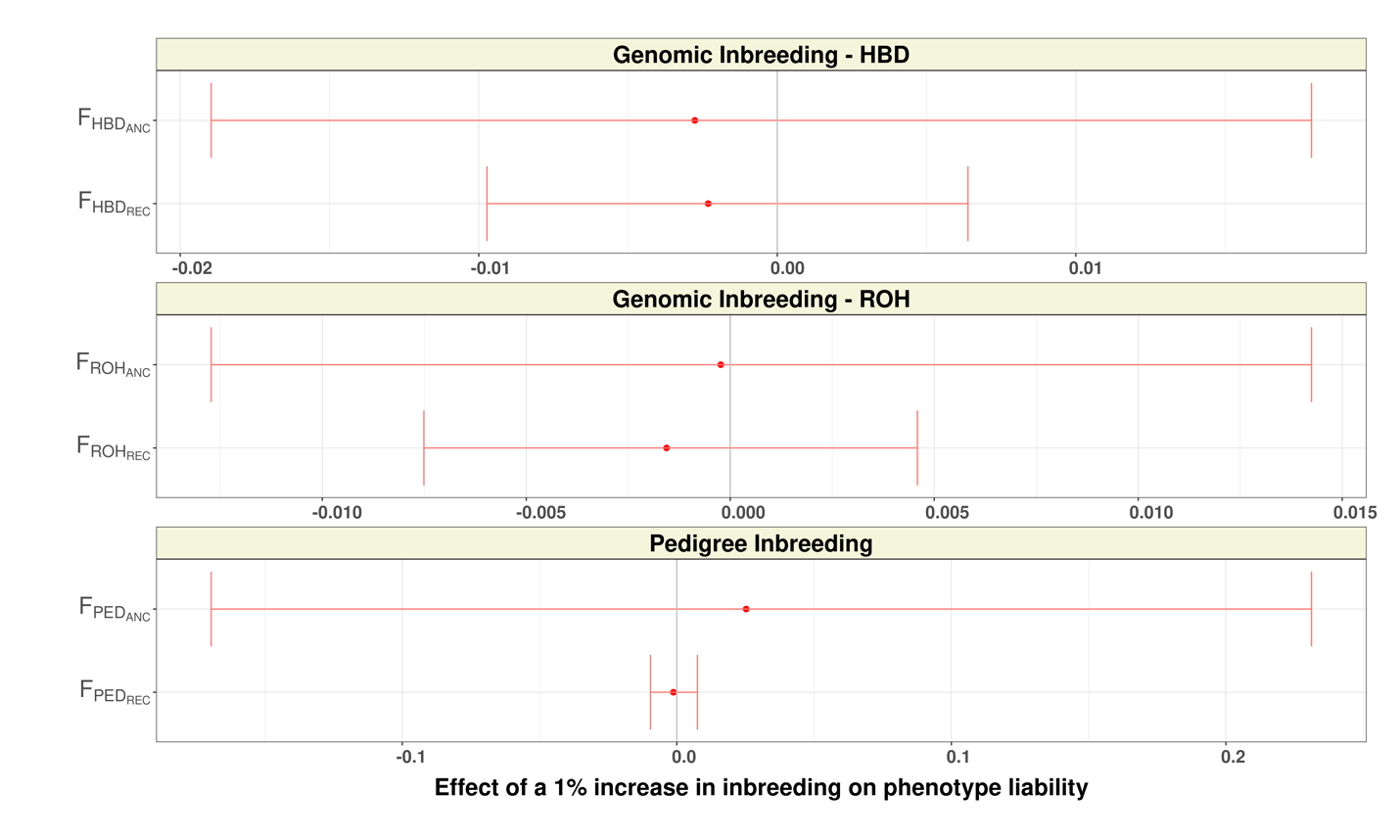

Supplement: Supplementary file 7 — Additional file 7: Figure S7. Effect of a 1% increase in recent and ancient inbreeding on heifer pregnancy. Estimates for the effect of a 1% increase in recent and ancient pedigree and genomic inbreeding. [file 12711_2021_644_MOESM7_ESM.docx]
